# Molecular basis for bacterial peptidoglycan recognition by LysM domains

**DOI:** 10.1038/ncomms5269

**Published:** 2014-06-30

**Authors:** Stéphane Mesnage, Mariano Dellarole, Nicola J. Baxter, Jean-Baptiste Rouget, Jordan D. Dimitrov, Ning Wang, Yukari Fujimoto, Andrea M. Hounslow, Sébastien Lacroix-Desmazes, Koichi Fukase, Simon J. Foster, Michael P. Williamson

**Affiliations:** 1 Krebs Institute, University of Sheffield, Firth Court, Western Bank, Sheffield S10 2TN, UK; 2 Department of Molecular Biology and Biotechnology, University of Sheffield, Firth Court, Western Bank, Sheffield S10 2TN, UK; 3 Centre de Biochimie Structurale, CNRS UMR 5048—UM 1—INSERM UMR 1054, F-34090 Montpellier, France; 4 INSERM, U872, Centre de Recherche des Cordeliers, Equipe 16, F-75006 Paris, France; 5 Université Pierre et Marie Curie, UMR-S 872, F-75006 Paris, France; 6 Université Paris Descartes, UMR-S 872, F-75006 Paris, France; 7 Department of Chemistry, Laboratory for Natural Products Chemistry, Osaka University, Osaka 560-0043, Japan; 8 These authors contributed equally to this work; 9 Present address: Institut Pasteur, Unité de Virologie Structurale, 28 Rue du Docteur Roux, F-75015 Paris, France

## Abstract

Carbohydrate recognition is essential for growth, cell adhesion and signalling in all living organisms. A highly conserved carbohydrate binding module, LysM, is found in proteins from viruses, bacteria, fungi, plants and mammals. LysM modules recognize polysaccharides containing *N*-acetylglucosamine (GlcNAc) residues including peptidoglycan, an essential component of the bacterial cell wall. However, the molecular mechanism underpinning LysM–peptidoglycan interactions remains unclear. Here we describe the molecular basis for peptidoglycan recognition by a multimodular LysM domain from AtlA, an autolysin involved in cell division in the opportunistic bacterial pathogen *Enterococcus faecalis*. We explore the contribution of individual modules to the binding, identify the peptidoglycan motif recognized, determine the structures of free and bound modules and reveal the residues involved in binding. Our results suggest that peptide stems modulate LysM binding to peptidoglycan. Using these results, we reveal how the LysM module recognizes the GlcNAc-X-GlcNAc motif present in polysaccharides across kingdoms.

## Abstract (web summary)

Proteins containing LysM domains recognize polysaccharides such as chitin and peptidoglycan, the main components of fungal and bacterial cell walls. Here the authors describe the molecular interactions between peptidoglycan and a LysM domain from the opportunistic bacterial pathogen *Enterococcus faecalis*.

## Graphical abstract

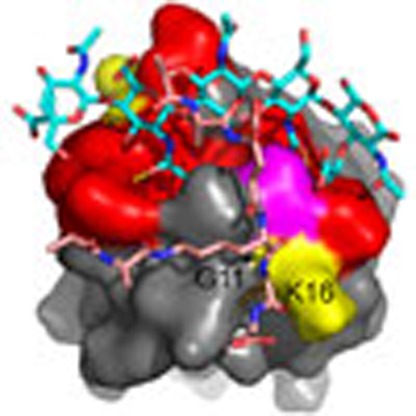

Molecular recognition of carbohydrates regulates essential biological processes in living organisms: cell development and differentiation^1^, response to bacterial and viral infection^2^, cell adhesion during inflammation and cancer metastasis^3^, modulation of the immune response^4^,^5^,^6^ and signalling^7^. One carbohydrate binding module (CBM) conserved across all kingdoms is LysM. It is present in bacterial extracellular proteins including hydrolases, adhesins and virulence factors such as Protein A from *Staphylococcus aureus*^8^. It is also found in proteins produced by fungal pathogens acting as modulators of host immunity^9^,^10^, and is present in a large number of proteins from insects, mammals and plants involved in defence against pathogens^11^,^12^,^13^,^14^ and symbiotic signalling^15^,^16^. LysM modules consist of 43–50 amino acids that adopt a highly conserved βααβ-fold, with particularly high sequence conservation in the first 16 residues^8^,^17^. Prokaryotic LysM modules bind peptidoglycan, the main component of the bacterial cell wall, made of alternating *N*-acetylglucosamine (GlcNAc) and *N*-acetylmuramic acid (MurNAc) residues, substituted by short peptide stems^18^. In eukaryotes, LysM domains have been shown to bind mainly to chitin, a β-1,4-linked GlcNAc polymer that is the main constituent of fungal cell walls^5^,^11^,^19^,^20^,^21^, as well as to peptidoglycan^19^. LysM receptors have recently been shown to bind directly to nodulation (Nod) factors produced by nitrogen-fixing bacteria, essential for symbiotic interaction^22^. Nod factors are lipooligosaccharides consisting of 3–5 β-1,4-linked GlcNAc residues *N*-acylated at their non-reducing end and decorated by several modifications^23^. Interestingly, LysM proteins produced by plants can bind two major components of bacterial and fungal pathogen cell walls (peptidoglycan and chitin, respectively) and activate a common signal transduction pathway leading to an immune response^19^.

Despite a wealth of knowledge as to the role of LysM proteins, only scarce information is available concerning the mechanism underpinning LysM–carbohydrate interaction^24^,^25^,^26^. The recent elucidation of the structure of *Arabidopsis thaliana* AtCERK1 LysM domain in complex with a chitooligosaccharide revealed the molecular basis for chitin recognition^25^. This work identified a complex network of hydrogen bonds between the main chain of the LysM modules and the acetyl groups of GlcNAc residues. Previous studies have addressed the role of bacterial multimodular domains in peptidoglycan binding^27^,^28^, but no functional study concerning the molecular basis of this interaction is available.

Here, we investigate how LysM domains bind to peptidoglycan, using as a model the multimodular LysM domain of *Enterococcus faecalis* AtlA, a peptidoglycan hydrolase with *N*-acetylglucosaminidase activity involved in cell division^29^,^30^. Interestingly, our results suggest that unlike the eukaryotic LysM domains from plants and fungi studied so far^25^,^26^,^31^,^32^ tandem bacterial LysM modules do not form any stable quaternary structure, and contribute to binding in an additive manner. Using peptidoglycan fragments and commercially available polysaccharides, we show that LysM recognizes both the GlcNAc moiety of polysaccharides and to a lesser extent the peptide stems. We present the solution structure of LysM, identify the LysM residues involved in polysaccharide binding and calculate a structure for the complex. On the basis of our results, we discuss the molecular basis for the promiscuous binding of LysM to polysaccharides including chitin, peptidoglycan and Nod factors.

## Results

### Multiple LysM modules provide additive binding potential to the domain

**Figure 1:**
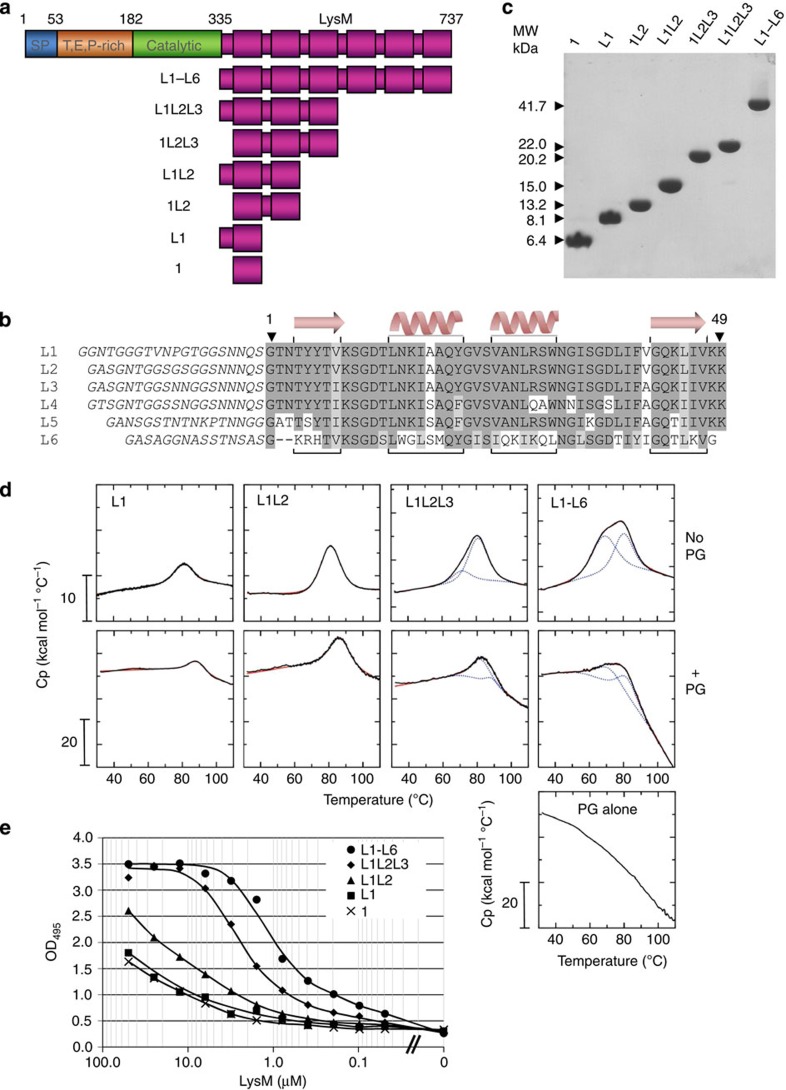
Contribution of LysM modules (1–6) and linker sequences (L) to the folding and binding activity of the LysM domain. (**a**) Domain organization of *E. faecalis* glucosaminidase AtlA and LysM-derived polypeptides studied. SP, signal peptide; T,E,P-rich, N-terminal domain of unknown function rich in threonine, glutamic acid and proline residues. Amino acid numbers refer to the transition between modules. (**b**) Sequence alignment of the six LysM modules present in the C-terminal domain of AtlA. Numbering refers to residues corresponding to the LysM module (49 residues); linker sequences are in italics. Identical amino acids in at least four modules are in dark grey boxes, conserved amino acids are in light grey boxes. Secondary structures determined by NMR for 1LysM are indicated. (**c**) SDS–PAGE of purified recombinant LysM polypeptides described in **a**; the molecular weight of each purified polypeptide is indicated. (**d**) Differential scanning calorimetry (DSC) profiles of recombinant LysM polypeptides in the absence (No PG) and presence of peptidoglycan (+PG). Red and dotted blue lines are theoretical curves corresponding to a two-state or a three-state unfolding, respectively. (**e**) Detection of peptidoglycan binding activities of LysM domains harbouring one to six LysM modules by ELISA.

The LysM domain of *E. faecalis* AtlA (Fig. 1a) contains six conserved LysM modules displaying 65–100% similarity to each other (Fig. 1b). Each module is preceded by a low complexity sequence of 16–23 residues, henceforth referred to as a linker region. Six variants containing 1–6 LysM modules, with or without a linker sequence at the N terminus, were overexpressed and purified (Fig. 1c; Supplementary Fig. 1). Differential scanning calorimetry (DSC) was used to investigate the respective contributions of LysM modules and linkers to the structural organization of the LysM domain, both in the absence and in the presence of peptidoglycan sacculi. For all constructs, change in heat capacity associated with protein unfolding revealed a reversible denaturation without significant aggregation (Fig. 1d). LysM domains made of one or two modules (1, L1, 1L2 and L1L2) presented two-state unfolding mechanisms, similar to the *Escherichia coli* MltD LysM domain^33^. Surprisingly, LysM variants with three and six modules showed an additional unfolding transition at lower temperatures, suggesting that increasing the size of the LysM domain was associated with the existence of a folding intermediate. This folding intermediate was also observed by circular dichroism spectroscopy (Supplementary Fig. 2). In keeping with these results, high pressure fluorescence experiments^34^ showed similar centres of spectral mass in all constructs tested, consistent with a similar environment of residue W31 (Supplementary Fig. 3). Dynamic light scattering experiments revealed a positive correlation between the hydrodynamic diameter and the number of modules in the constructs analysed (Supplementary Fig. 4). Altogether, these results therefore suggest that quaternary interactions are not responsible for the folding intermediate. Aside from the additional transition observed for L1L2L3 and L1–L6, all variants present a melting temperature (Tm) at ~80 °C indicating that the presence of multiple tandem LysM modules had no major impact on the thermostability of the domain (Table 1). This observation suggests that a particular number of LysM modules is not required to form a stable domain. Interestingly, addition of a linker sequence at the N terminus of the constructs with 1, 2 and 3 modules was systematically associated with a moderate Tm increase (+1.6 °C for L1, +0.7 °C for L1L2 and +0.9 °C for L1L2L3) and a significant increase in enthalpy change (Δ*H=*29 kcal mol^−1^ for L1, 30 kcal mol^−1^ for L1L2 and 11 kcal mol^−1^ for L1L2L3) (Table 1) indicating a stabilizing effect of the N-terminal linker. DSC analyses of LysM domains bound to peptidoglycan sacculi revealed similar DSC profiles for all constructs with a systematic Tm increase due to LysM–peptidoglycan interaction (Table 1) and Δ*H* values increasing with both the number of LysM modules and linkers in an additive manner. Altogether, these results suggest that multiple LysM modules do not adopt a particular quaternary structure to generate a functional protein. This conclusion remains valid whether LysM is bound to peptidoglycan or not.

The independence of LysM modules is supported by NMR analyses. Modules 1, 2 and 3 have almost identical sequences, whereas the linkers preceding them have a number of differences (Fig. 1b). ^15^N HSQC NMR spectra of labelled 1, L1, 1L2, L1L2, 1L2L3 and L1L2L3 constructs are almost superimposable, showing that there is no interaction between modules, or between modules and linkers (Supplementary Fig. 5). Furthermore, analysis of NMR chemical shifts of the linker regions using the random coil index^35^,^36^ and TALOS-N^36^ indicates that the linkers are disordered.

To further explore the contribution of LysM modules 1–6 to the binding activity of the full-length protein, we assayed by enzyme-linked immunosorbent assay (ELISA) the binding of constructs containing variable numbers of LysM modules to immobilized peptidoglycan (Fig. 1e). All the constructs bound peptidoglycan in a dose-dependent manner. A single motif was sufficient to bind peptidoglycan, and the presence of a linker sequence at the N terminus of the minimal single LysM construct had no noticeable impact on binding activity. Binding increased with the number of LysM modules, indicating an additive contribution of LysM modules to binding. These results are consistent with the DSC and NMR analyses, suggesting that LysM modules do not interact with each other, either when free in solution or when binding to peptidoglycan, and thus behave as beads on a string rather than generating a quaternary structure essential for binding.

The lack of interaction between LysM domains on binding is seen most clearly by NMR titration of the 1L2L3 construct with GlcNAc_6_ (Supplementary Fig. 6). The changes seen in the spectrum on addition of GlcNAc_6_ are almost identical to those seen on addition of GlcNAc_6_ to a single LysM module (discussed in more detail below). Furthermore, the changes in the NMR spectrum show a very similar dependence on ligand concentration, demonstrating that the affinity of each of the three modules in 1L2L3 for GlcNAc_6_ is similar to that of the single module of construct 1 (Fig. 1a).

### Analysis of peptidoglycan properties required for binding

**Figure 2:**
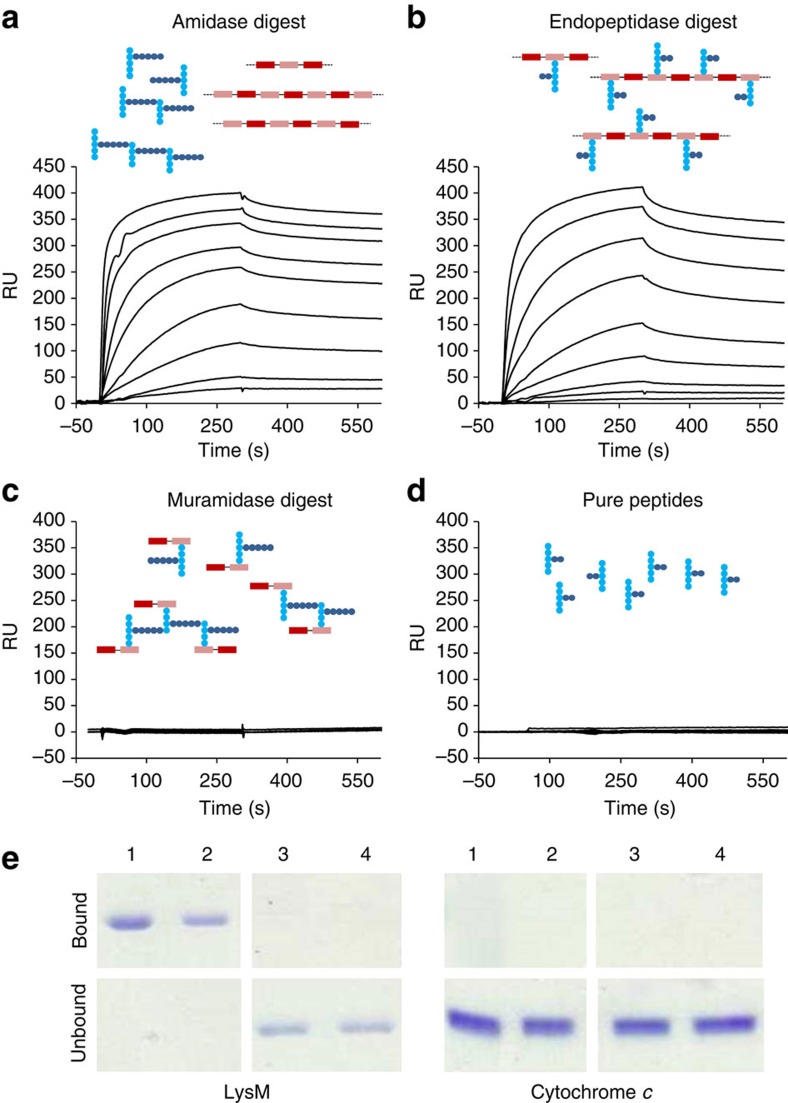
Identification of the structural motif recognized by LysM. Red rectangle is GlcNAc, pink rectangle is MurNAc, blue circles are peptide stem with (darker blue) crosslinking peptides. The six LysM module polypeptide (L1–L6) was immobilized on a CM5 chip and binding was measured with surface plasmon resonance (SPR) using analyte mixtures corresponding to soluble *Staphylococcus aureus* peptidoglycan fragments generated using three enzymes with distinct cleavage specificities: (**a**) amidase digest, containing a mixture of peptide stems and glycan chains; (**b**) endopeptidase digest, containing linear (non cross-linked) peptidoglycan; (**c**) muramidase digest, containing disaccharides linked to peptide stems, some of which are cross-linked; (**d**) synthetic peptide stems. RU, resonance units (**e**) Affinity purification of AtlA L1–L6 LysM domain and cytochrome *c* with insoluble polysaccharides: 1, peptidoglycan; 2, chitin; 3, cellulose; 4, xylan. Protein remaining in the supernatant (Unbound) and associated with the pellet (Bound) were analysed by SDS–PAGE and Coomassie staining. Cytochrome *c* was used as a control, as this protein displays a similar isoelectric point to the LysM domain (pI=9.6 versus 10.06, respectively). No binding activity was detected with cytochrome *c* using any of the polysaccharides.

Binding of LysM modules to peptidoglycan fragments was studied using surface plasmon resonance (SPR). Recombinant LysM proteins made of six modules were immobilized on a sensor chip and binding was assayed using analyte mixtures prepared from peptidoglycan sacculi hydrolysed by enzymes displaying distinct cleavage specificities (Fig. 2). Dose-dependent binding was observed with soluble peptidoglycan fragments resulting from amidase or endopeptidase digestion (Fig. 2a,b). By contrast, binding was abolished when peptidoglycan was digested with a muramidase (mutanolysin) or when synthetic peptide stems were assayed (Fig. 2c,d). Taken together, these results indicate that integrity of the glycan strands is essential for LysM–peptidoglycan interaction and that the peptide stem is not a sufficient motif for binding.

The glycan binding activity of LysM was further analysed using other insoluble polysaccharides (Fig. 2e; Supplementary Fig. 7). LysM showed affinity for peptidoglycan and chitin but not for cellulose or xylan, suggesting that the amide group at the C_2_′ position of the GlcNAc residues is essential for binding.

LysM binds to both peptidoglycan and chitin. However, the kinetics of binding to these two ligands are strikingly different. Binding to chitin displays rapid on and off rates (Supplementary Fig. 8), whereas binding to peptidoglycan has much slower on and off rates (Fig. 2).

### Identification of the minimal motif recognized by LysM

Chitin oligosaccharides were used to compare the impact of multiple modules on binding activity and define the minimal length of the carbohydrate recognized by LysM (Table 2). Steady-state/equilibrium SPR analyses (Supplementary Fig. 8) showed that a single LysM module consisting of 49 amino acids was sufficient to bind chitooligosaccharides. The presence of a linker sequence at the N terminus of the single module did not have any impact on binding: GlcNAc_5_ gave apparent K_D_ values of 12.3±1.4 μM and 12.1±1.4 μM for polypeptides 1 and L1, respectively. In agreement with ELISA assays using immobilized peptidoglycan sacculi (Fig. 1e), apparent binding affinity increased with the number of LysM modules (Table 2). For all oligosaccharides tested, apparent K_D_ values varied only additively (sixfold maximum) when the number of LysM modules increased from 1 to 6. For example, with GlcNAc_5_, K_D_ values varied from 12.1±1.4 μM (one module) to 2.2±0.3 μM (six modules). This limited increase in apparent affinity is expected for relatively short oligosaccharides, which are likely to be recognized by a single module. Similar binding for all LysM domains suggested that a particular number of LysM modules is not required to generate a functional binding domain.

A minimum of three GlcNAc residues were required for detectable binding to a single LysM module, whereas four residues showed close to optimal binding (Table 2). The estimated affinities increased with oligosaccharide chain length, with apparent K_D_ values of 407.9±80.8 μM, 43.4±3.0 μM, 12.3±1.4 μM and 6.0±0.8 μM for GlcNAc_3_, GlcNAc_4_, GlcNAc_5_ and GlcNAc_6_, respectively, suggesting that effective binding requires at least four GlcNAc residues. These results are in agreement with the absence of binding when disaccharide-peptides produced by muramidase (mutanolysin) digestion were used as analytes (Fig. 2c).

NMR measurements of affinity between a single LysM module and oligosaccharides confirmed the same trends, though with a tendency to weaker affinities (Supplementary Table 1). NMR confirmed that the minimum saccharide length needed for effective binding is four residues, with little or no increase in affinity on going to longer oligosaccharides; binding to xylan, cellulose and chitosan (de-acetylated chitin) oligomers was not detectable. The requirement for acetylation of N_2_′ (that is, lack of binding to chitosan) is not surprising and has been well documented^37^,^38^.

### NMR structure of AtlA LysM

**Figure 3:**
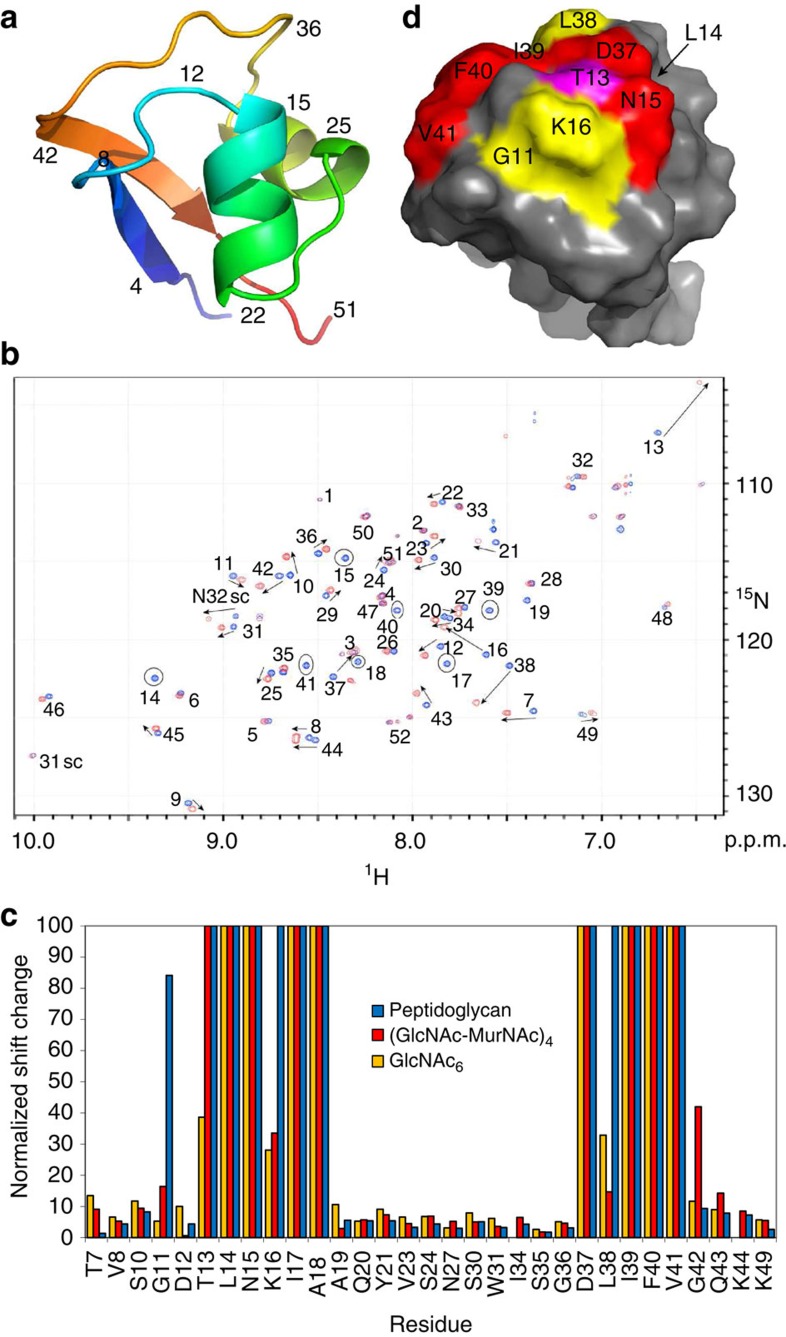
The 1LysM module and its interaction with carbohydrates. (**a**) NMR structure of 1LysM. β-Sheets are residues T4-V8 and G42-V47; α-helices are residues L14-Y21 and V25-N32. (**b**) ^15^N HSQC NMR spectra of 1LysM, free (blue) and saturated with 44 equivalents of GlcNAc_6_ (red). Residues showing significant chemical shift changes are indicated by arrows, and residues broadened and not reappearing by the end of the titration are circled. (**c**) Summary of chemical shift changes observed for the titration of 1LysM with peptidoglycan (blue), octasaccharide (GlcNAc-MurNAc)_4_ (red) and GlcNAc_6_ (yellow). Results for GlcNAc_5_ and GlcNAc_4_ are very similar to those for GlcNAc_6_, whereas tetrasaccharide (GlcNAc-MurNAc)_2_ is similar to octasaccharide. The sugar backbone is glucosamine for all three oligosaccharides, but in octasaccharide and peptidoglycan alternate sugars are *N*-acetyl muramic acid (that is, bearing a lactate group at O_3_′), while in peptidoglycan most *N*-acetyl muramic acids also carry a peptide stem. Thus, effects of the lactate are seen as differences between GlcNAc_6_ and the other two, whereas effects of the peptide stem are seen as differences between peptidoglycan and the other two. Residues broadened in the titrations and not observed by the end of the titration are shown as bars with a normalized shift change of 100. Residues are not shown for which any shift change is smaller than the mean. (**d**) Representation of the data shown in **c**. Residues broadened beyond detection are in red, residue T13 implicated in binding the *N*-acetyl muramic acid lactate group is in magenta and residues implicated in binding peptide stems are in yellow.

The first LysM module of AtlA (construct 1 in Fig. 1a and 1LysM in Supplementary Fig. 1) was expressed in *E. coli* doubly labelled with ^15^N and ^13^C isotopes. A complete resonance assignment was obtained using standard triple resonance experiments. Structures were calculated using ^1^H–^1^H NOE-derived distance restraints, ^3h^*J*_NC′_ scalar couplings obtained from a two-dimensional long-range H(N)CO experiment^39^ (which turned out to be an excellent way to identify specific inter-residue backbone hydrogen bonds; Supplementary Fig. 9), and dihedral angle restraints obtained using TALOS-N^36^ (Fig. 3a). Members of the structural ensemble have few violations and overlay with a backbone RMSD of 0.56 Å (Supplementary Table 2, Supplementary Figs 10 and 11). The fold comprises four regions of secondary structure: β-strand (T4-V8), α-helix (L14-Y21), α-helix (V25-N32), β-strand (G42-V47) and is similar to that of previously determined LysM modules (Supplementary Fig. 12). Ramachandran analysis of φ and ψ dihedral angles shows that 89% of the residues are in the most favoured region and 11% of residues lie in the additionally allowed region^40^. An extensive network of hydrogen bonds stabilizes the protein, including an unusual buried asparagine, N32, whose sidechain makes hydrogen bonds to two backbone carbonyls (Supplementary Fig. 13).

### The LysM–peptidoglycan complex

To identify the LysM residues involved in peptidoglycan binding, NMR titrations were carried out using chemically defined oligosaccharides (Fig. 3b) and soluble peptidoglycan fragments consisting of glycan chains with peptide stems attached to the MurNAc residues produced by treatment of *S. aureus* cell walls with lysostaphin^41^ (Fig. 2b). A considerable degree of broadening was detected during the NMR titrations, particularly for the residues most in contact with the ligand. For many of these residues, the backbone NH signal disappeared completely, reappearing in some cases with a large excess of ligand. This behaviour can indicate either slow-to-intermediate exchange between free and bound states, or conformational change in the protein associated with binding^42^. The chemical shift changes seen in the titration are close to linear but not completely (Supplementary Fig. 14), strongly suggesting the presence of conformational equilibria associated with binding, involving small populations of conformationally altered proteins^43^. Line broadening has also been observed in earlier studies of LysM^24^,^26^, suggesting that structural rearrangement is a common feature of LysM modules. ^13^C HSQC titrations also showed extensive broadening during the titration, involving the same residues as seen by ^15^N HSQC experiments, but in addition, including residues within the interior of the protein such as L28 and I39 (adjacent to the buried residues I17 and A18, which also experience large changes in ^13^C shift). Because ^13^C chemical shifts are affected mainly by conformational change rather than direct effects of ligand binding^42^,^43^, these results confirm that there is some conformational rearrangement of the protein on binding to the oligosaccharides, which extends from the binding interface towards the centre of the protein.

These rearrangements and line broadening do not preclude analysis of binding and measurement of affinity using NMR, which was therefore carried out (Supplementary Fig. 15, Supplementary Table 1). Two groups of residues were most affected by titration with peptidoglycan fragments (blue bars in Fig. 3c): T13-A18, located in the loop between strand 1 and helix 1 and at the N terminus of helix 1; and D37-V41, located between helix 2 and strand 2 (Fig. 3c,d). These residues form a contiguous surface that was previously shown to mediate interaction with chitin^25^,^26^, therefore suggesting that peptidoglycan binding by LysM occurs via a mechanism common to all LysM modules so far analysed at the molecular level.

To determine which parts of the intact peptidoglycan interact with the protein 1LysM, titration experiments were repeated with a synthetic octasaccharide, (GlcNAc-MurNac)_4_, lacking peptide stems and chitooligosaccharides GlcNAc_4–6_, lacking both peptide stems and the lactyl groups. In titration experiments with octasaccharide (red bars in Fig. 3c), broadening associated with G11, K16 and L38 drastically decreased compared with the titrations with peptidoglycan fragments, suggesting that these amino acids interact with peptide stems. As these residues (the yellow residues in Fig. 3d) are located on both sides of the binding site, our result suggests that the peptide stem can have variable geometry, at least in the conformationally unrestricted ligands used here. Finally, comparison between chemical shift changes associated with the binding of octasaccharide (red bars in Fig. 3c) and GlcNAc_6_ (orange bars in Fig. 3c) revealed that only T13 interacts significantly with the lactyl groups of the MurNAc residues. This result locates a lactyl group close to T13 (Fig. 3d). Titrations of the T13A mutant with GlcNAc_6_, octasaccharide and peptidoglycan fragments show that the broadening of T13 with octasaccharide and peptidoglycan is no longer seen in the T13A mutant, confirming this interaction.

**Figure 4:**
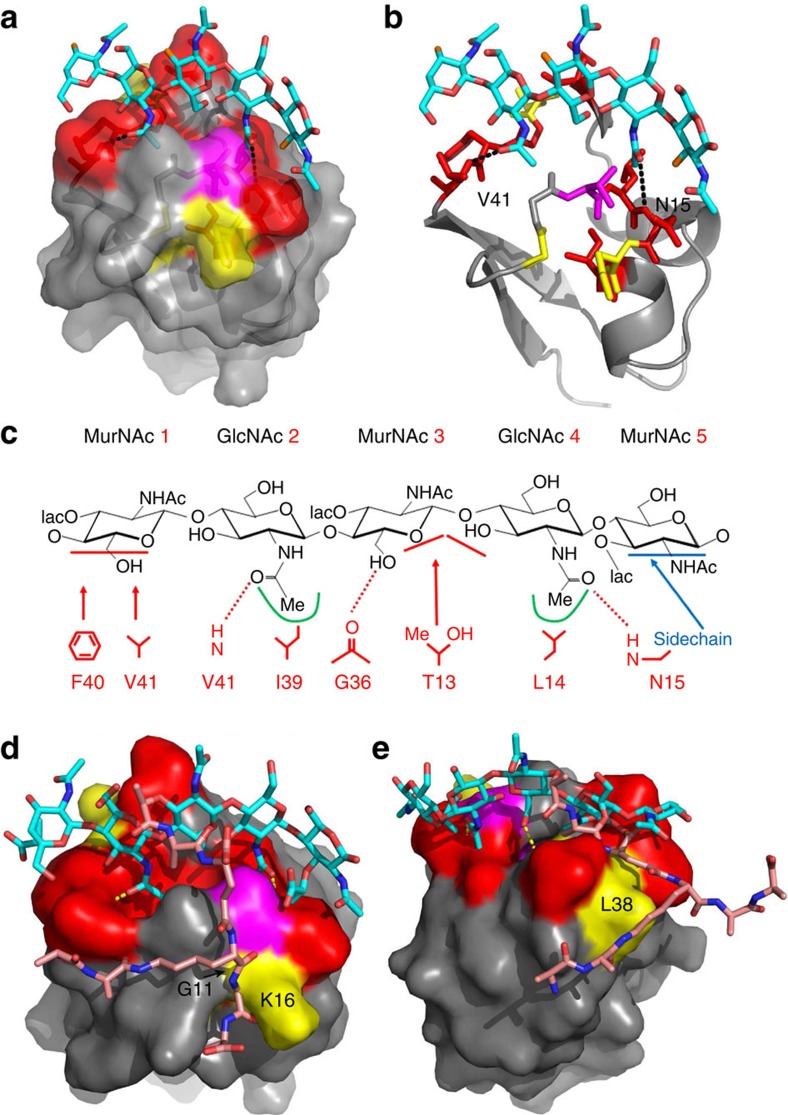
Model of the structure of 1LysM bound to GlcNAc_5_. (**a**) Partially transparent protein surface indicated, showing the groove along the surface, with deeper pockets to accommodate the *N*-acetyl groups. The O_3_′ atoms where the MurNAc peptide stems would be attached in a peptidoglycan ligand are shown in orange. (**b**) Identical view, without the protein surface. Sidechain atoms are shown for T13 and F40. The colour schemes in **a** and **b** are the same as in Fig. 3d. (**c**) Key interactions with MurNAc-(GlcNAc-MurNAc)_2_. Hydrogen bonds are shown by dashed lines. The horizontal bars indicate that the hydrophobic interaction is to the face of a sugar ring. The two pockets are indicated by green lines. Also shown in blue is the binding location involving N15 discussed in the text which may be important for distinguishing between chitin and peptidoglycan. As discussed in the text, residues I17 and A18 (which show large changes in ^15^N HSQC; Fig. 3) are buried and do not interact directly with the ligand, but move because of conformational rearrangement. The shift change seen for D37 HN is due to the interaction with G36 carbonyl. (**d**,**e**) Model of the interaction between 1LysM and peptidoglycan with peptide stem. The peptide stem is shown in two possible orientations, either side of the glycan backbone, interacting either with G11/K16 or with L38.

On the basis of these results, a structure of the complex was determined by docking GlcNAc_5_ onto the solution structure of 1LysM. As expected, the protein structure rearranges slightly on binding (r.m.s. backbone change=1.3 Å, mainly at residues G36-I39), forming a pocket within which one of the GlcNAc *N*-acetyl groups is buried. The oligosaccharide binds edge-on in a groove on the protein surface, with the *N*-acetyl groups from GlcNAc sugar residues 2 and 4 forming hydrogen bonds to backbone amide groups of residues V41 and N15, respectively (Fig. 4a,b). Sugar 1 (on the left of Fig. 4a) stacks against the aromatic ring of F40, and the 6′-hydroxyl of sugar 3 makes a hydrogen bond to the carbonyl group of G36, leaving the 3′ position (where the peptide stem is attached) pointing away from the protein surface. Interactions with the protein are shown in Fig. 4c. The structure of the complex is fully consistent with the titration and binding data. It suggests possible locations for the peptide stems, which can be located in shallow grooves on either side of the glycan backbone (Fig. 4d,e).

The binding site identified here is similar to that identified in earlier studies^24^,^25^,^26^,^32^, implying that the binding mode is conserved among LysM modules that bind to peptidoglycan, chitin and Nod factors. Furthermore, interactions were observed with lactyl groups and with the peptide stems (not present in chitin and Nod factors), which suggests that the LysM modules of AtlA have evolved to bind peptidoglycan. NMR shows that LysM undergoes a slow conformational rearrangement on binding peptidoglycan, presumably required to ensure optimal recognition of peptidoglycan.

### Mutational analysis of the LysM domain

To confirm the NMR mapping of residues involved in binding, 11 alanine single-site substitution mutants of 1LysM were analysed. ^15^N HSQC spectra of all the mutants (Supplementary Figs 16 and 17) revealed amide signals in similar positions and as well-dispersed as those of wild-type 1LysM, suggesting that none of the mutants has major conformational differences. Binding affinities of alanine mutants to GlcNAc_5_ were measured by SPR (Table 3). By far the largest effects seen were for T13A, L14A and I39A, which reduced affinity by factors of 60, 80 and 80, respectively. D37A and L38A reduced the affinity approximately 12-fold, whereas D12A, N15A, K16A and F40A had only a small effect (0- to 6-fold reduction in apparent affinity).

### Peptide stems modulate LysM binding activity

To further explore the LysM–peptidoglycan interaction, binding of a single LysM module (1LysM) to various defined ligands was analysed by NMR. Two tetrasaccharides, (MurNAc-GlcNAc)_2_ and (GlcNAc-MurNAc)_2_, bound 1LysM with relatively low affinities (K_D_ values >1 mM and 650 μM, respectively) as compared with GlcNAc_4_ (K_D_=90 μM), thus suggesting that the lactyl group (which is present in MurNAc but not GlcNAc) inhibits binding, possibly by interacting unfavourably with T13. As previously shown for chitooligosaccharides, binding affinity increased with the length of the glycan chains (K_D_=80 μM for (GlcNAc-MurNAc)_4_). Remarkably, synthetic tetrasaccharides harbouring a peptide stem attached to the lactyl group of the MurNAc residues bound less tightly to 1LysM than their counterparts with no peptide stem. Although the low affinity and the limited availability of the ligands tested did not allow us to carry out titrations with a large excess of substrates, we were able to determine K_D_ values of 1.8±0.5 mM and >1 mM for (GlcNAc-MurNAc-dipeptide)_2_ or (GlcNAc-MurNAc-heptapeptide)_2_, respectively. Altogether, the use of synthetic and purified peptidoglycan fragments indicated that the peptide stems act as negative discriminants that modulate, but do not prevent, binding.

### Towards a common mechanism underpinning LysM–carbohydrate interaction

A summary of binding interactions between AtlA 1LysM and peptidoglycan strands is shown schematically in Fig. 4c. Key interactions are made with the *N*-acetyl groups of the GlcNAc-X-GlcNAc moiety, in which the methyl groups fit into hydrophobic pockets while the carbonyls form hydrogen bonds. Important interactions are located at the bottom edge of the central sugar, in particular with the 6′-hydroxyl group, however, no interactions have been identified involving the top edge. Thus, there is little discrimination between MurNAc and GlcNAc sugar residues in this central position. Only one stacking interaction involving the aromatic ring of F40 against the ring of sugar residue 1 has been identified. However, mutation of F40 to alanine had no large effects on affinity (Table 3) and the interaction is not optimal as F40 has considerable space in which to move, giving rise to unfavourable entropy changes on binding. Thus, the binding interaction is unusual in having no strongly required aromatic group in the binding site^44^. There are sidechain interactions with most of the amino acids that are well conserved among LysM modules. We conclude that LysM primarily recognizes GlcNAc-X-GlcNAc (X=GlcNAc or MurNAc), with further specificity (for example discrimination between peptidoglycan, chitin and Nod factors) determined by secondary factors, as discussed below.

## Discussion

LysM domains are found in virtually all living organisms (except Archaea) and bind polymers containing GlcNAc residues^8^. In this work, we provide a detailed study of the structure/function relationships of a LysM domain that binds peptidoglycan, the major component of the bacterial cell wall.

Previous reports on eukaryotic LysM modules suggested that disulphide bond formation is a posttranslational modification essential for carbohydrate recognition^31^,^32^. Structural data of *A. thaliana* CERK1 showed that the three individual modules are tightly packed against each other. In *Pteris ryukyuensis* PrChi-A, the interaction between the two modules, mediated via disulphide bridges, is crucial for optimal stability of the domain. In both the fungal chitin binder Ecp6 (ref. 45)^45^ and the rice chitin-elicitor binding protein CEBiP^46^, recognition of chitin requires close interaction between two or more LysM modules to afford specific and tight binding of chitin. By contrast, prokaryotic LysM domains usually do not contain cysteine residues^8^ and no information is available concerning the role of multiple modules in their structural organization and activity. We therefore sought to identify potential formation of quaternary structures using a combination of DSC and NMR. Our results strongly support the idea that individual LysM modules do not form organized quaternary structures, either free or bound to polysaccharide ligands. Therefore the existence of multiple independent binding modules may confer flexibility to the domain, a property likely to optimize binding to a complex three-dimensional substrate such as peptidoglycan. The presence of a variable number of modules in LysM proteins (1–6) contrasts with other cell wall binding domains such as the S-layer homology domain^47^ or the conserved domain PF04122 (ref. 48), which are always made of three modules. In the case of the S-layer homology domain, structural data showed that three modules are required to generate a functional binding site^49^. It seems likely that the compact multimodular structures seen for the eukaryotic disulphide-bonded proteins *A. thaliana* CERK1, *Medicago truncatula* NFP, *P. ryukyuensis* PrChi-A, *Cladosporium fulvum* Ecp6 and rice CEBiP made of several modules are not typical in prokaryotes.

We have shown that multiple LysM modules increase the binding to peptidoglycan (Fig. 1e and Table 2) when either short oligosaccharides or intact sacculi were used as ligands. Interestingly, increasing the number of LysM modules has a more significant impact on affinity when peptidoglycan was used as a ligand, as compared with chitooligosaccharides. This therefore suggests that individual LysM modules bind in a cooperative manner to long glycan chains present in cell walls, but not to short oligosaccharides. Similar conclusions were drawn in two studies on the *N*-acetylglucosaminidase AcmA from *Lactococcus lactis*^27^ and the D,L-endopeptidase CwlS from *Bacillus subtilis*^28^. Although one can expect a cooperative effect of multiple modules, a detailed study of the impact of LysM multimodularity on binding represents a major challenge. Access to detailed kinetic analyses is currently not possible due to the lack of a defined substrate available in sufficient amounts. The minimal motif recognized by a single LysM module consists of four carbohydrates (Table 2). In a recent study, Wong *et al.* reported the binding of *B. subtilis* CwlS LysM domain to disaccharide-peptides linked via their peptide stem (dimers, trimers and tetramers)^28^. The molecular basis for this interaction between LysM domains and glycan chains made of two carbohydrate residues remains unexplained. It contrasts with our results indicating that AtlA LysM domain does not bind to disaccharide-peptide mixtures including monomers, dimers, trimers and tetramers (Fig. 2c).

On the basis of structural and binding properties, a simple classification of CBMs into three types has been proposed^44^: type A, surface-binding CBMs; type B, glycan-chain binding CBMs; and type C, small sugar binding CBMs. Several lines of evidence suggest that LysM modules belong to type B CBMs. Unlike crystalline cellulose, which forms a relatively flat surface recognized by type A CBMs, chitin oligosaccharides and peptidoglycan strands display a helical structure^50^,^51^. Accordingly, the recent three-dimensional structure of *A. thaliana* CERK1 bound to a chitopentaose revealed that the sugar backbone binds in a shallow groove formed by the loops between strand 1-helix 1 and helix 2-strand 2 of the protein^25^. Although some aromatic residues have been shown to contribute to binding (for example, Y72 in *P. ryukyuensis* PrChi-A, F40 in *E. faecalis* AtlA), stacking interactions have a limited role^25^. In the present study, we showed that only one of the five aromatic residues present in each LysM module interacts with the ligand. Substitution of the corresponding residue (F40) by an alanine retains 25% of wild-type binding activity. One hallmark of LysM binding to carbohydrates is an important hydrogen bond network that results from interactions with main chain atoms, ensuring a contact surface with four sugars that represents a minimal binding motif. In agreement with this property, we have shown that alanine mutations have only a limited impact on binding activity (Table 3).

Chemical shift changes observed for a LysM module upon binding chemically defined oligosaccharides revealed line broadening, a phenomenon that can be attributed to conformational adjustment, which is confirmed by comparing free and oligosaccharide-bound 1LysM structures (Figs 3d and 4a). This property was also described for two other LysM domains^24^,^26^, and is an original property for CBMs, which usually bind in fast exchange with very small structural change^44^. Conformational change on ligand binding to CBMs is typically observed for conformationally flexible saccharides^52^, suggesting that LysM may need to accommodate a range of peptidoglycan conformations as found in bacterial cell walls.

LysM ligands are structurally related polysaccharides containing GlcNAc residues (Supplementary Fig. 7), and this amino sugar was therefore suggested to be essential for binding^8^. Recent crystallographic data revealed the presence of two well-defined pockets that accommodate *N*-acetyl groups from two GlcNAc residues separated by one pyranose, confirming the importance of this functional group^25^. This finding is confirmed in the present study.

Different LysM modules have been reported to bind to chitin (polymers of GlcNAc), peptidoglycan (polymers of [GlcNAc-MurNAc]) and Nod factors, which are short oligosaccharides characterized by having hydrophobic substitutions at C_2_′, C_4_′ and C_6_′ of the non-reducing terminal sugar^32^. Recognition of Nod factors requires a leucine as the residue facing C_3_′ of this sugar, which is L118 in the second LysM module of *Lotus japonicus* Nod factor receptor 5 (ref. 32). In AtlA LysM, the residue in the corresponding spatial position (though not in sequence) is F40, which promotes the binding of an extended polymer rather than a terminal sugar; aromatic residues are also found in corresponding positions in several chitin-binding proteins^24^,^25^. In the case of chitin recognition, an aromatic residue has been shown to have an important role^26^. The absence of an aromatic residue at the equivalent position in AtlA LysM (N15) allows stacking against the face of a MurNAc residue in peptidoglycan. Altogether, these results suggest that recognition of peptidoglycan is promoted both by having an aromatic residue binding the non-reducing end of the ligand and a non-aromatic residue in the binding cleft mediating interactions at the reducing end of the ligand (F40 and N15, respectively in AtlA LysM). Although *E. faecalis* AtlA is a *bona fide* peptidoglycan hydrolase dedicated to septum cleavage during division^29^,^30^, our results unexpectedly show that it binds chitin with a higher affinity than peptidoglycan. However, SPR analyses showed that binding to these two polymers is associated with distinctly different kinetics (compare Fig. 2 and Supplementary Fig. 8). A major determinant explaining the weaker affinity of AtlA LysM for peptidoglycan is the presence of peptide stems, likely due to steric hindrance. An alternative explanation is that LysM binds with higher affinity to peptidoglycan stretched under turgor in intact cell walls, a property that has previously been suggested as a factor controlling autolytic activities^53^. Of course, one cannot rule out the possibility that promiscuous LysM binding might permit an undiscovered environmental role.

Qualitative assays revealed that prokaryotic LysM domains bind peptidoglycan structures containing various peptide stem compositions and cross-links^18^. Here, we explore LysM binding to defined peptidoglycan fragments and provide evidence that although the peptide stem is not necessary for binding, it interacts with several residues and modulates binding affinity as a negative discriminant. Access to pure peptidoglycan fragments in sufficient amounts is a major challenge to further explore binding specificity of LysM domains and study the impact of their properties on binding (for example, size, amino acid sequence, isoelectric point of individual repeats and length of the intermodule sequence). Pursuing LysM structure/function studies is a first step towards the engineering of LysM domains with altered ligand repertoires. It has direct implications for the development of novel strategies using LysM domains to target therapeutic molecules to pathogens.

## Methods

### Bacterial strains, plasmids and growth conditions

Strains and plasmids used in this study are described in Supplementary Table 3. For protein purification, *E. coli* was grown at 37 °C in Luria-Bertani broth or in M9 minimal medium containing 1 g l^−1 15^NH_4_Cl (and 3 g l^−1 13^C_6_-glucose where necessary), supplemented with 150 μg ml^−1^ ampicillin. When the cultures had reached an optical density of 0.6 at 600 nm, production of the recombinant proteins was induced by addition of 1 mM isopropyl-β-D-thiogalactopyranoside, and incubation continued for 16 h.

### Purification of recombinant LysM polypeptides

The sequences of the recombinant LysM polypeptides overexpressed and purified are described in Supplementary Fig. 1. Induced cells were harvested and resuspended in buffer A (50 mM Tris-HCl (pH 8.0) containing 300 mM NaCl), and crude lysates were obtained by sonication (5 × 30 s, 20% output; Branson Sonifier 450). Proteins were loaded onto Ni^2+^ nitrilotriacetate agarose resin (Qiagen GmbH, Hilden, Germany), washed with 5 mM imidazole in buffer A, and eluted with 500 mM imidazole in buffer A. Recombinant His-tagged proteins were further purified by size-exclusion chromatography on a Superdex75 HR 26/60 column (Amersham Biosciences, Uppsala, Sweden). For DSC experiments, the column was equilibrated with 50 mM sodium acetate (pH 5.0); for NMR and SPR experiments, proteins were purified using 40 mM Na_2_HPO_4_ (pH 6.0). The fractions were analysed by SDS–PAGE, pooled and concentrated down to 50 μM for both NMR and DSC experiments. Protein concentration was estimated by measuring absorbance using the theoretical extinction coefficient calculated by the Expasy Protparam tool ( http://web.expasy.org/protparam/).

### Polysaccharide affinity purification

The affinity of the complete AtlA LysM domains (L1–L6 in Fig. 1a) for various polysaccharides was determined by incubating 32 μg of protein with 1 mg of crab shell chitin (Sigma, Ref. C-7170), cellulose (Sigma, Ref. 8002) or xylan (Sigma, Ref. X-0502) in a total volume of 150 μl of phosphate-buffered saline (PBS; 137 mM NaCl, 2.7 mM KCl, 10 mM Na_2_HPO_4_, 2 mM KH_2_PO_4_, pH 7.4). After 30 min of gentle rocking at ambient temperature, the insoluble fraction was harvested by centrifugation (5 min, 20,000 *g*) and 50 μl of the supernatant was collected (unbound fraction). The remaining supernatant was discarded and the insoluble fraction was washed three times with 1.5 ml of PBS. The washed pellet was resuspended in 150 μl of Laemmli buffer (bound fraction) and boiled for 5 min. Five microliters of the unbound and bound fractions were loaded on a 13% (w/v) SDS–PAGE and stained with Coomassie brilliant blue.

### Peptidoglycan purification

Peptidoglycan sacculi were extracted from exponentially growing *S. aureus* cells with boiling SDS as previously described^30^. After treatment with pronase and trypsin, peptidoglycan-bound polymers were removed by incubation in fluorhydric acid 48% (v/v) at 4 °C for 48 h. Pure peptidoglycan was extensively washed with distilled water and freeze-dried. For ELISA experiments (see below), sacculi were diluted at a concentration of 10 mg ml^−1^ and broken in the presence of acid-washed glass beads (Sigma, Ref. G4649) using a fast-prep machine (MPBio) for six cycles of 30 s at maximum speed, with 2 min pauses between cycles.

### Peptidoglycan digestion for SPR interaction assays

Peptidoglycan sacculi were digested with well-characterized enzymes displaying distinct peptidoglycan cleavage specificity: mutanolysin, a muramidase, (Sigma, Ref. M9901); lysostaphin, a glycyl-glycyl endopeptidase (Sigma, Ref. L7386) or the amidase domain from *S. aureus* Atl autolysin^54^. One milligram of peptidoglycan was digested in a final volume of 250 μl. Specific buffers and enzyme amounts were as follows: mutanolysin, 50U in 20 mM phosphate buffer (pH 6.0); lysostaphin, 50 μg in 25 mM phosphate buffer (pH 7.5); Atl amidase, 75 μg in 10 mM Tris–HCl (pH 7.0) containing 1 mM CaCl_2_. After centrifugation for 20 min at 22,000 *g*, two-fold serial dilutions of the digestion mixtures in PBS (1/200 to 1/51,200) were used to test interaction with AtlA 1LysM domain by SPR. Muropeptide analysis of the digestion mixture by rp-HPLC confirmed the heterogeneity of the peptidoglycan fragments solubilised by the enzymes (Supplementary Fig. 18).

### Enzyme-linked immunosorbent assay

Ninety-six-well polystyrene plates (Nunc Maxisorp) were coated with 100 μl of pure peptidoglycan fragments diluted to 10 mg ml^−1^ in PBS. After incubation for 1 h at 22 °C, the plates were washed three times in PBS-Tween-20 (0.25% v/v; PBS-T) and residual binding sites were blocked with 300 μl of 2% (m/v) skimmed milk in PBS-T for 1 h at 22 °C. After three washes in PBS-T, recombinant LysM domains serially diluted in PBS-T were added to peptidoglycan-coated plates and incubated for 1 h at 22 °C. After three washes in PBS-T, the plates were incubated with a rat anti-LysM antibody raised against the first LysM module of AtlA (‘1 LysM’ in Supplementary Fig. 1) diluted 1/3,000 in PBS-T containing 2% (m/v) skimmed milk in PBS-T. After 1 h at 22 °C, the plate was washed five times with PBS-T and incubated with a peroxidase-conjugated goat anti-rat IgG (SIGMA Ref. A9037) diluted 1/3,000 in PBS-T containing 2% (m/v) skimmed milk in PBS-T for 1 h at 22 °C. Immunoreactivity of IgG was revealed by measuring the absorbance at 492 nm after addition of peroxidase substrate (0.4 mg ml^−1^ o-phenylenediamine dihydrochloride, 0.4 mg ml^−1^ urea hydrogen peroxide and 50 mM phosphate-citrate, pH 5.0; Sigma-Aldrich). All reactions were stopped by addition of 2 N H_2_SO_4_ after the same incubation time.

### Differential scanning calorimetry

LysM samples at a concentration of 50 μM were extensively dialysed using a 3.5 kDa molecular weight cut-off membrane (SPECTRUM Labs) at 4 °C against 50 mM sodium acetate (pH 5.0), and degassed before DSC measurements (MicroCal VP-DSC calorimeter). The dialysis buffer was used as a heat reference. Each sample was measured twice in the 30–110 °C range at 1 °C min^−1^ rate to corroborate folding reversibility, which was found >85% for all LysM samples. Individual experiments were carried out at least twice for each sample. To study protein unfolding in the presence of peptidoglycan, protein samples were incubated with 10 mg ml^−1^ of pure peptidoglycan sacculi and the mixture was dialysed, as for unbound LysM samples. To help LysM–peptidoglycan binding, the second buffer change was performed at 20 °C. Under these conditions, >95% of LysM proteins were bound to peptidoglycan. Samples were then degassed, loaded and measured as for the unbound samples. As reported previously^55^, the DSC profile of the peptidoglycan sacculi alone reveals a prominent negative slope of irreversible nature. In all cases, baseline corrections were performed by subtracting the corresponding buffer profile. Data were analysed using the MicroCal Origin software using the provided two-state unfolding model for 1, L1, 1L2, L1L2 proteins and three-state unfolding model for 1L2L3, L1L2L3 and L1–L6 proteins. Plots were represented using ProFit (QuantumSoft).

### Circular dichroism

The experiments were carried out in 50 mM sodium acetate buffer (pH 5.0) using 50 μM protein samples at a temperature rate of 1 °C min^−1^ controlled by a Peltier adapted to Chirascan CD spectrometer (Applied Photophysics).

### Fluorescence under pressure

50 μM protein samples in 50 mM sodium acetate buffer (pH 5.0) were excited at 280 nm and the fluorescence emission spectra was recorded between 305 and 450 nm at 20 °C using an ISS steady-state fluorimeter (Champaign, IL). High pressure perturbation and data analysis were carried out as described previously^34^. In brief, LysM samples were subjected to pressure from 1 bar to 3 kbar in 0.2 kbar intervals. All spectra were recorded at equilibrium and analysed by calculating the centre of spectral mass using ProFit software (QuantumSoft). The centre of spectral mass is not significantly affected by the number of LysM modules or by the effect of pressure. This is indicative of an unaltered tryptophan (W31) chemical environment within the module, suggesting the absence of quaternary structure among LysM variants^56^.

### Dynamic light scattering

All measurements were recorded in a Zetasizer Nano S DLS device (Malvern Instruments) in 50 mM sodium acetate (pH 5.0) at 20 °C using 50 μM protein samples.

### Preparation of sensor surfaces

LysM polypeptides were immobilized on CM5 sensor chips using a standard amine coupling method at a flow rate of 10 μl min^−1^. Briefly, the carboxymethylated dextran surface was activated by injecting 40 μl of a solution containing 0.2 M 1-ethyl-3-[3-(dimethylamino)propyl]carbodiimide hydrochloride (EDC; Sigma) and 0.05 M *N*-hydroxysuccinimide (Sigma). Covalent immobilization of LysM polypeptides was carried out using 70 μl of a protein solution diluted in 5 mM maleate buffer (pH 5.0). The reaction was quenched with 40 μl of 1 M ethanolamine-HCl (pH 8.5).

### SPR experiments

SPR experiments were performed on a BIACORE 2000 instrument with research grade CM5 sensor chips at a temperature of 25 °C. The running buffer was HEPES-buffered saline (HBS-P; 10 mM HEPES (pH 7.4), 150 mM NaCl, 0.005% (v/v) Tween-20). If not otherwise indicated, the flow rate was 10 μl min^−1^. The chip was regenerated using a 1-min pulse of 6 M guanidine hydrochloride followed by an injection of 30 μl of HBS-P buffer. Various immobilization densities and flow rates were tested to define optimal steady-state analysis conditions (Supplementary Fig. 8).

### NMR

NMR experiments were conducted on Bruker DRX-800 and DRX-600 (plus cryoprobe) spectrometers at 25 °C in 40 mM phosphate buffer (pH 6.0). Assignments were made with *Asstools*^57^ using standard triple resonance experiments on a double labelled sample of L1 LysM and were transferred to other constructs on the basis of the high similarity of shifts. Ligand titrations were carried out on 50 μM protein solutions using SOFAST-HMQC^58^. The final titration had a volume up to twice the original, but no concentration-dependent effects were observed. K_D_ values were obtained from fitting to standard saturation curves using Microsoft Excel. Chemical shift changes were analysed as a weighted sum of ^1^H and ^15^N shift changes: shift=
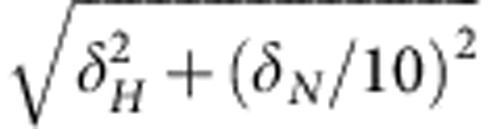
 (ref. 42). For the structure calculation, NOEs were measured from a ^15^N- and ^13^C-edited 100-ms 3D NOESY spectrum, and hydrogen bonds were identified through the direct observation of inter-residue ^3h^*J*_NC′_ scalar couplings in a two-dimensional long-range H(N)CO experiment^39^. Spectra were processed and analysed using TOPSPIN (versions 1.3 and 2.1) and FELIX 2007 software (Felix NMR, Inc., San Diego, CA). One hundred structures were annealed from randomised starting coordinates, refined using the dynamic annealing protocol within CNS version 1.21 (ref. 59), and further refined with TIP3P explicit water molecules^60^. The 10 lowest energy structures were chosen to represent the structural ensemble and the coordinates and NMR chemical shifts have been deposited in the RCSB Protein Data Bank ( www.pdb.org) and the BioMagResBank ( www.bmrb.wisc.edu) under PDB code 2mkx and accession number 19799, respectively.

### Docking

Docking was carried out using the HADDOCK approach^61^ with residues 12, 13, 15, 38 and 40 defined as the ‘active’ residues, based on titration data and surface accessibility. Standard parameters were used, except that electrostatics were turned off during the initial rigid docking; intermolecular interactions were very weak during rigid docking and scaled up later; 1,000 structures were calculated, of which 200 were refined in explicit water, using 2,500 steps at 300 K followed by 600 cooling steps. Following extensive docking trials to locate the sugar carbonyls properly into the binding pockets and prevent the ligand rotating to end up side-on to the protein (to maximize buried surface area), hydrogen bond restraints between the GlcNAc carbonyls and the amide groups of residues V41 and N15 were added as unambiguous restraints.

### Synthesis of peptidoglycan fragments

Tetrasaccharide (GlcNAc-β−1,4-MurNAc)_2_ and octasaccharide (GlcNAc-β−1,4-MurNAc)_4_ fragments of peptidoglycan were synthesized on the basis of a previously reported method^62^. Final products were systematically analysed by NMR and mass spectrometry. Briefly, appropriately protected (GlcNTroc(β1-4)MurNTroc)-trichloroacetoimidate donor and the (GlcNTroc(β1-4)MurNTroc) glycosyl acceptor (liberated at the 6-position of the non-reducing end of GlcNTroc) were reacted with trimethylsilyl trifluoromethanesulfonate (TMSOTf ) in dry CH_2_Cl_2_ with MS4A at −15 °C for 10 min. After quenching the reaction, the crude compound was purified by silica-gel flash chromatography (toluene:EtOAc=5:1) to give fully protected tetrasaccharide or octasaccharide compounds. The ester protection of the carboxylic acids at the MurNAc moieties was cleaved with LiOH in dioxane:THF:H_2_O (2:4:1), and then all benzyl-type protections were cleaved under H_2_ (20 atm) with Pd(OH)_2_/C in acetic acid for 1 day. The synthetic compounds were purified by rp-HPLC using a 10 × 250 mm Cosmosil C18 AR300 column (Nacalai Tesque, Inc.). After equilibration in water-TFA (0.1% (v/v)), tetrasaccharide or octasaccharide were eluted using an acetonitrile gradient (1 to 70% (v/v) in 45 min) and freeze-dried.

## Author contributions

S.M. and M.P.W. designed the research. S.M. constructed LysM expression vectors and purified recombinant proteins for DSC, NMR and SPR. M.D. and J.-B.R. carried out DSC experiments. J.D.D. and S.M. performed SPR analyses. M.P.W. performed NMR experiments and assigned the LysM HSQC spectrum with the help of A.M.H. N.J.B. and M.P.W. calculated the NMR structures and analysed NMR data. N.W., Y.F. and K.F. provided synthetic peptidoglycan fragments. S.M., M.D., N.J.B., J.-B.R., J.D.D., S.L.-D., Y.F., S.J.F. and M.P.W. analysed the data. S.M. and M.P.W. wrote the paper.

## Additional information

**Accession codes:** Coordinates and NMR chemical shifts for the 10 lowest energy structures (out of 100) have been deposited in the RCSB Protein Data Bank (PDB) and the Biological Magnetic Resonance Data Bank (BioMagResBank, BMRB) under PDB accession code 2MKX and BMRB accession code 19799, respectively.

**How to cite this article:** Mesnage, S. *et al.* Molecular basis for bacterial peptidoglycan recognition by LysM domains. *Nat. Commun.* 5:4269 doi: 10.1038/ncomms5269 (2014).

## Supplementary Material

Supplementary Information Supplementary Figures 1-18, Supplementary Tables 1-3 and Supplementary References

## Figures and Tables

**Table 1:** DSC analysis of LysM domains bound or unbound to peptidoglycan.

**Protein**	**No peptidoglycan**				**Bound to peptidoglycan**			
	**Δ*****H***_**1**_ **(kcal mol**^−**1**^**)**	***Tm***_**1**_ **(°C)**	**Δ*****H***_**2**_ **(kcal mol**^−**1**^**)**	***Tm***_**2**_ **(°C)**	**Δ*****H***_**1**_ **(kcal mol**^−**1**^**)**	***Tm***_**1**_ **(°C)**	**Δ*****H***_**2**_ **(kcal mol**^−**1**^**)**	***Tm***_**2**_ **(°C)**
1	24.5±0.4	80.1±0.1	NA*	NA	ND	ND	ND	ND
L1	53.7±1.2	81.7±0.1	NA	NA	51.5±0.5	88.3±0.1	NA	NA
1L2	122.1±0.6	80.3±0.1	NA	NA	ND	ND	ND	ND
L1L2	152.6±2.4	81.0±0.1	NA	NA	180.0±1.0	86.4±0.1	NA	NA
1L2L3	50.6±10.1	74.3±0.6	106.7±10.2	81.0±0.3	ND	ND	ND	ND
L1L2L3	34.4±8.6	71.5±0.1	133.3±8.7	81.1±0.1	132.7±14.9	82.3±0.6	30.1±14.5	89.4±0.45
L1–L6	150.8±1.8	69.1±0.1	114.0±1.7	80.4±0.1	141.4±8.7	71.8±0.4	145.6±8.6	81.9±0.2

ND, not determined.

Differential scanning calorimetry (DSC) data were fitted to a two- or three-state unfolding model.

^*^NA: three-state unfolding model not applicable.

**Table 2:** Affinity constants of LysM domains for chitooligosaccharides.

	**GlcNAc**_**6**_	**GlcNAc**_**5**_	**GlcNAc**_**4**_	**GlcNAc**_**3**_	**GlcNAc**_**2**_
L1–L6	1.5±0.1	2.2±0.3	18.6±4.5	701.9±87.7	4,416±1,486
L1L2L3	3.0±0.1	4.6±0.4	22.3±1.8	627.7±32.8	7,100±4,842
L1L2	5.0±0.3	9.6±0.8	38.1±2.8	678.1±57.3	>5,000
L1	5.6±0.5	12.1±1.4	50.4±4.1	453.0±58.6	>5,000
1	6.0±0.8	12.3±1.4	43.4± 3.0	407.9±80.8	>5,000

Apparent K_D_ values of LysM domains containing different numbers of modules and linkers (L) are indicated in μM±s.d.

**Table 3:** Binding affinities of LysM alanine mutants determined by SPR.

**LysM**	**K**_**D**_ **(μM)**	**Residual binding (%)**
WT	11.7±1.7	100
D12A	72.0±15.2	16.2
T13A	730.2±83.1	1.6 (17.1)
L14A	927.9±151.1	1.3
N15A	21.5±3.9	64.1 (50.0)
K16A	13.1±1.2	89.3
N32A	15.8±2.3	74.0
D37A	138.3±14.2	10.0
L38A	145.5±27.7	12.4 (28.9)
I39A	1,021±224.2	1.3 (1.6)
F40A	54.0±12.0	25.5 (29.7)
V41A	11.7±2.1	100.0 (50.1)

GlcNAc_5_ was used as the analyte. Residual activity is the ratio of the apparent affinity to that of wild-type, obtained from surface plasmon resonance (SPR) or NMR (NMR values in brackets).
