# Supplementary material for: Molecular basis for bacterial peptidoglycan recognition by LysM domains
Source: Nat Commun. 2014 Jun 30;5:4269. doi: 10.1038/ncomms5269 (PMC4083421; doi:10.1038/ncomms5269)
Supplement: Supplementary Information — Supplementary Figures 1-18, Supplementary Tables 1-3 and Supplementary References [file ncomms5269-s1.pdf]

1 (1LysM)
*M***GTNTYYTVKSGDTLNKIAAQYGVSVANLRSWNGISGDLIFVGQKLIVKK**GSHHHHHH

L1
*MGGNTGGGTVNPGTGGSNNQS***GTNTYYTVKSGDTLNKIAAQYGVSVANLRSWNGISGDLIFVGQKLIVK**
KGSHHHHHH

1L2
*M***GTNTYYTVKSGDTLNKIAAQYGVSVANLRSWNGISGDLIFVGQKLIVKK***GASGNTGGSGSGGSNNNQS*
GTNTYYTVKSGDTLNKIAAQYGVSVANLRSWNGISGDLIFVGQKLIVKKGSHHHHHH

L1L2
*MGGNTGGGTVNPGTGGSNNQS***GTNTYYTVKSGDTLNKIAAQYGVSVANLRSWNGISGDLIFVGQKLIVK**
K*GASGNTGGSGSGGSNNNQS***GTNTYYTVKSGDTLNKIAAQYGVSVANLRSWNGISGDLIFVGQKLIVKK**
GSHHHHHH

1L2L3
*M***GTNTYYTVKSGDTLNKIAAQYGVSVANLRSWNGISGDLIFVGQKLIVKK***GASGNTGGSGSGGSNNNQS*
GTNTYYTVKSGDTLNKIAAQYGVSVANLRSWNGISGDLIFVGQKLIVKK*GSAGNTGGSNNGGSNNNQS***G**
TNTYYTIKSGDTLNKIAAQYGVSVANLRSWNGISGDLIFAGQKIIVKKRSHHHHHH

L1L2L3
*MGGNTGGGTVNPGTGGSNNQS***GTNTYYTVKSGDTLNKIAAQYGVSVANLRSWNGISGDLIFVGQKLIVK**
K*GASGNTGGSGSGGSNNNQS***GTNTYYTVKSGDTLNKIAAQYGVSVANLRSWNGISGDLIFVGQKLIVKK**
*GSAGNTGGSNNGGSNNNQS***GTNTYYTIKSGDTLNKIAAQYGVSVANLRSWNGISGDLIFAGQKIIVKK**RS
HHHHHH

L1-L6
*MGGNTGGGTVNPGTGGSNNQS***GTNTYYTVKSGDTLNKIAAQYGVSVANLRSWNGISGDLIFVGQKLIVK**
K*GASGNTGGSGSGGSNNNQS***GTNTYYTVKSGDTLNKIAAQYGVSVANLRSWNGISGDLIFVGQKLIVKK**
*GSAGNTGGSNNGGSNNNQS***GTNTYYTIKSGDTLNKIAAQYGVSVANLRSWNGISGDLIFAGQKIIVKK***GT*
*SGNTGGSSNGGSNNNQS***GTNTYYTIKSGDTLNKISAQFGVSVANLQAWNNISGSLIFAGQKIIVKK***GANS*
*GSTNTNKPTNNGG***GATTSYTIKSGDTLNKISAQFGVSVANLRSWNGIKGDLIFAGQTIIVKK***GASAGGNAS*
*STNSASG***KRHTVKSGDSLWGLSMQYGISIQKIKQLNGLSGDTIYIGQTLKVG**GSHHHHHH

Supplementary Figure 1. Amino acid sequences of the recombinant LysM polypeptides overexpressed and purified in this study. For each construct, the sequence of LysM modules (1-6, as defined in Fig. 1b) appears in red, while the sequences corresponding to linker regions (L) are in italics. All constructs harbour a C-terminal Histidine tag.

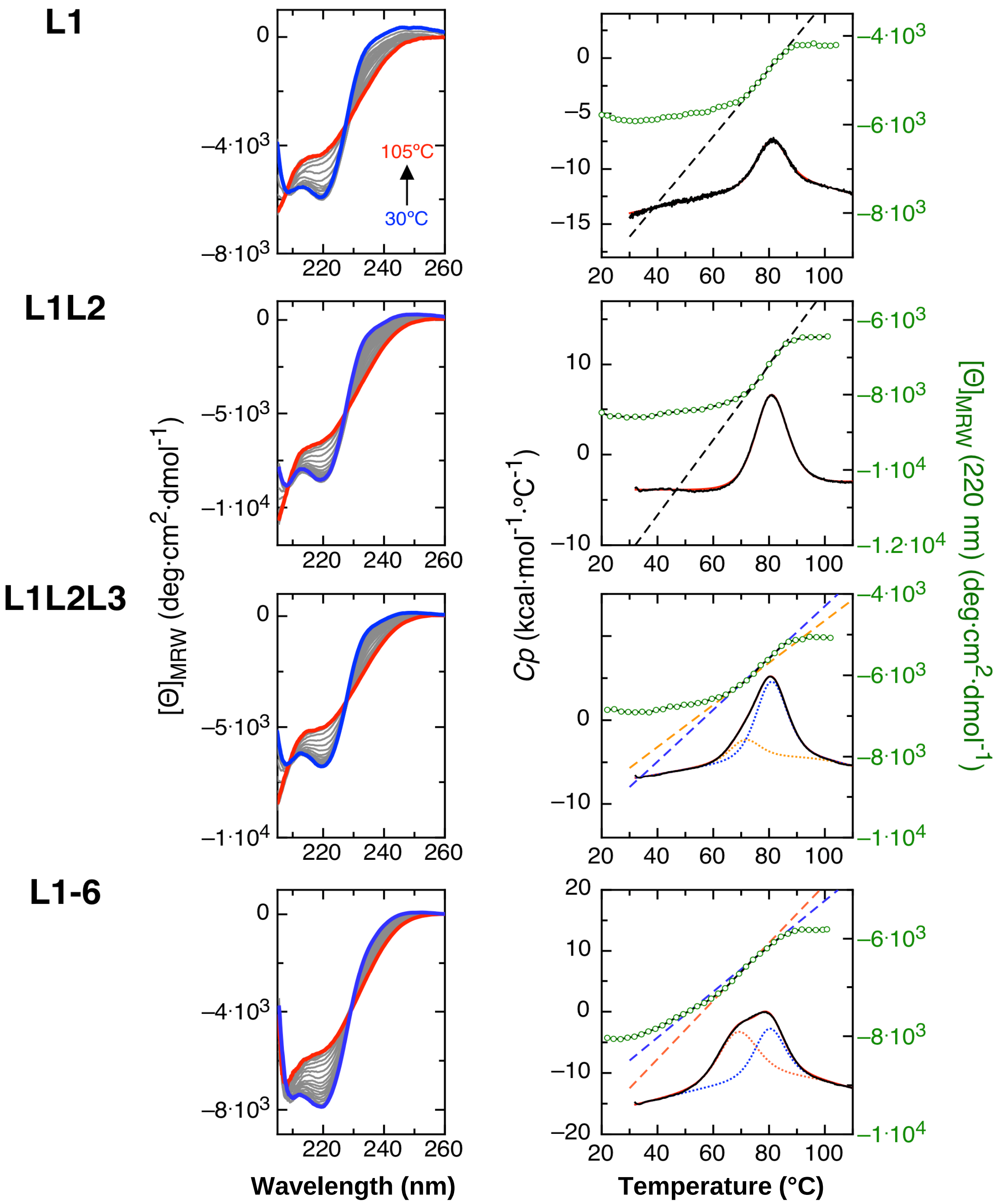

Supplementary Figure 2 . Comparison of the thermal unfolding profile of LysM domains followed by circular dichroism (CD) and differential scanning calorimetry (DSC). Left column: thermal dependence of CD spectra of LysM variants L1, L1L2, L1L2L3 and L1-L6. Right column: CD signals followed at 220 nm (green) and DSC profiles shown in Figure 1d of the main text. Dashed lines on the CD signal are linear regression fits to experimental data at the Tm ± 4ºC plotted to visualize the correspondence between CD and DSC profiles. CD measurements were reversible and were repeated at least twice. The correspondence between the CD signal and DSC profiles corroborates a three state unfolding model for L1L2L3 and L1-L6, whilst L1 and L1L2 variants unfold according to a two-state model.

a

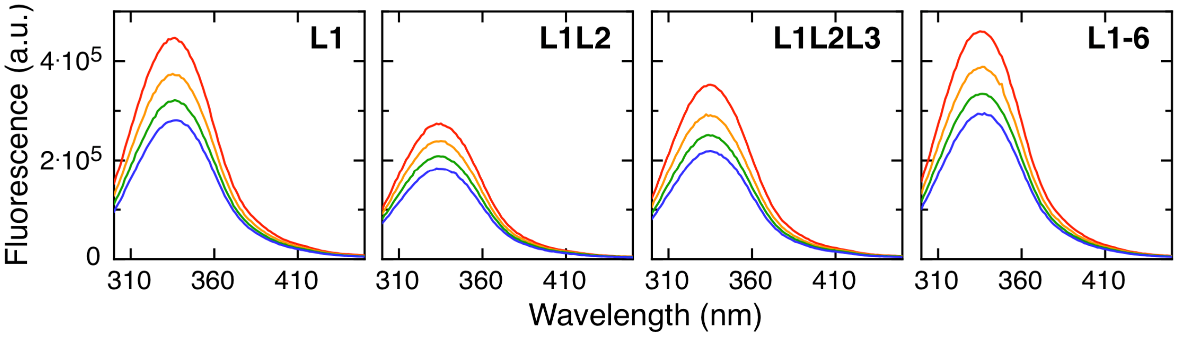

b

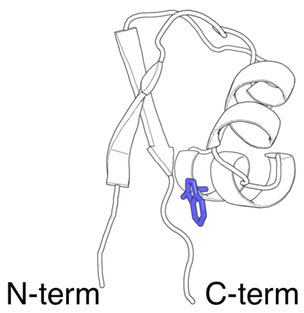

c

Protein	L1	L1L2	L1L2L3	L1-L6
Pressure (bar)	Center of Spectral mass (nm)			
1	340.9	339.9	340.3	340.7
1000	341.0	339.9	340.3	340.9
2000	341.3	340.1	340.5	341.1
3000	341.6	340.2	340.7	341.4

Supplementary Figure 3. Negligible effect of high pressure on LysM variants. (**a**) Fluorescence emission spectra of L1, L1L2, L1L2L3 and L1-L6 proteins recorded at 1 bar (red), 1 kbar (yellow), 2 kbar (green) and 3 kbar (blue). (**b**) Solution structure of 1LysM (PDB code: 2mkx), the position of the single tryptophan residue (W31) is represented in blue sticks. (**c**) Centre of spectral mass of LysM variants as a function of pressure.

a

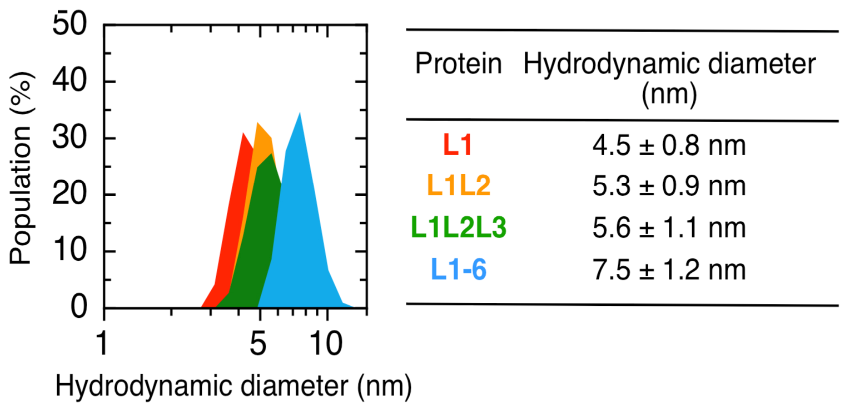

Protein	Hydrodynamic diameter (nm)
L1	4.5 ± 0.8 nm
L1L2	5.3 ± 0.9 nm
L1L2L3	5.6 ± 1.1 nm
L1-6	7.5 ± 1.2 nm

b

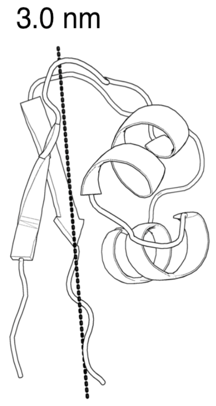

Supplementary Figure 4. Size distribution of LysM domains measured by dynamic light scattering. (**a**) Hydrodynamic diameter of LysM variants. The table shows the maximum and standard deviation of the size distributions calculated from a fit to a gaussian function. (**b**) Solution structure of 1LysM (PDB code: 2mkx), the dashed line represents the largest end-to-end distance in the molecule. Considering the presence of the N-terminal 20 amino acid loop (Fig. 1b) of L1, the measured hydrodynamic diameter of L1 agrees with the expected value as estimated from the LysM solution structure. The presence of multiples LysM modules increases the hydrodynamic diameter distribution in a non-additive manner, in agreement with the flexible nature of the connecting loops.

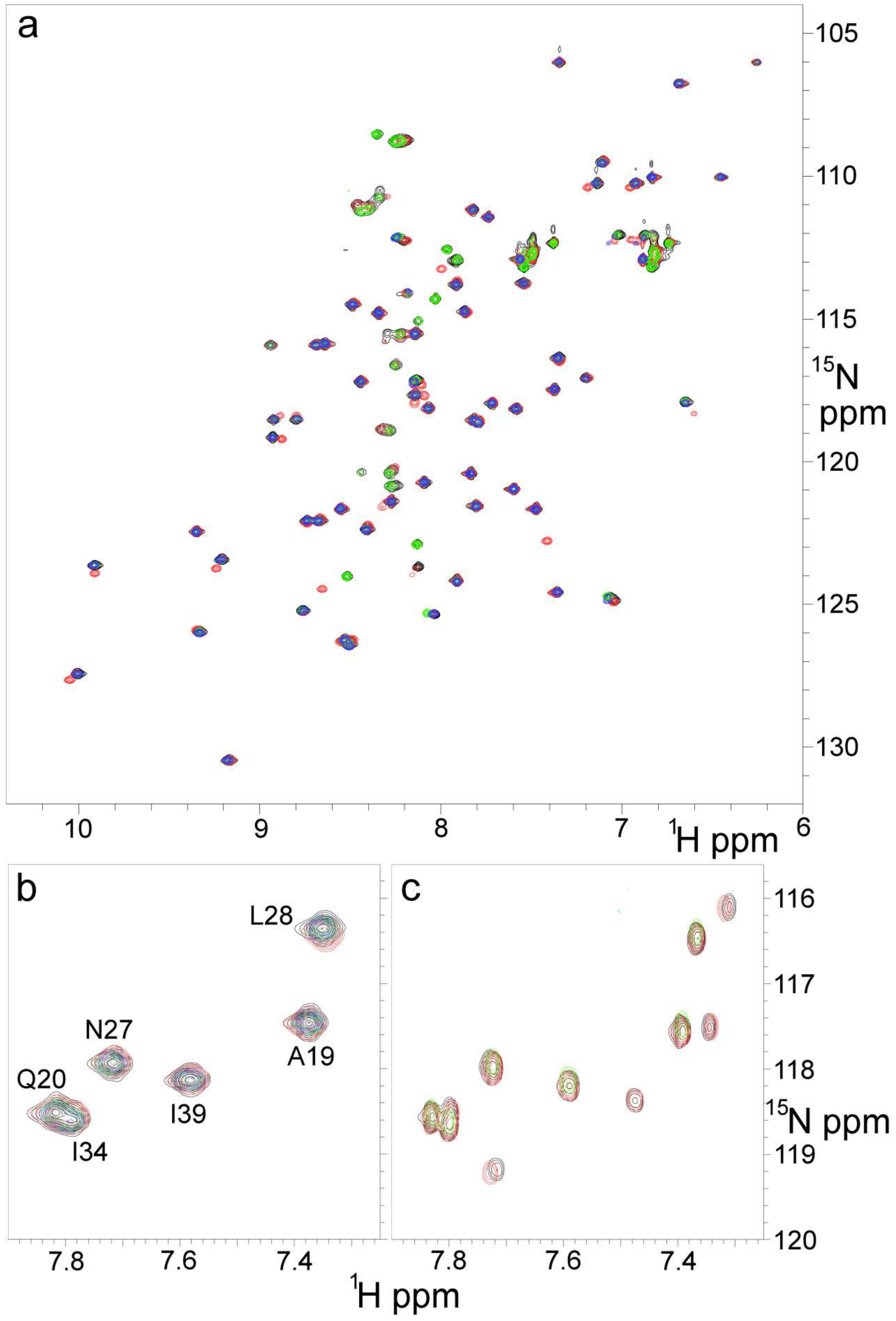

Supplementary Figure 5. Comparison of ^{15}N HSQC spectra of single and multiple LysM modules. (**a**) Overlay of 1 (blue), L1 (green), 1L2 (red) and L1L2 (black). Signals from linker residues are evident at random coil positions (^1H ca. 8.2 ppm). All residues superimpose except for signals in and around the β-sheet in one module in 1L2, indicating a local rearrangement of the sheet. (**b**) Expansion of (**a**). (**c**) Same region as in (**b**), for 1 (green), 1L2L3 (red) and L1L2L3 (black). Two of the three modules superimpose exactly and one has signals slightly shifted, partly because of sequence differences in module 3.

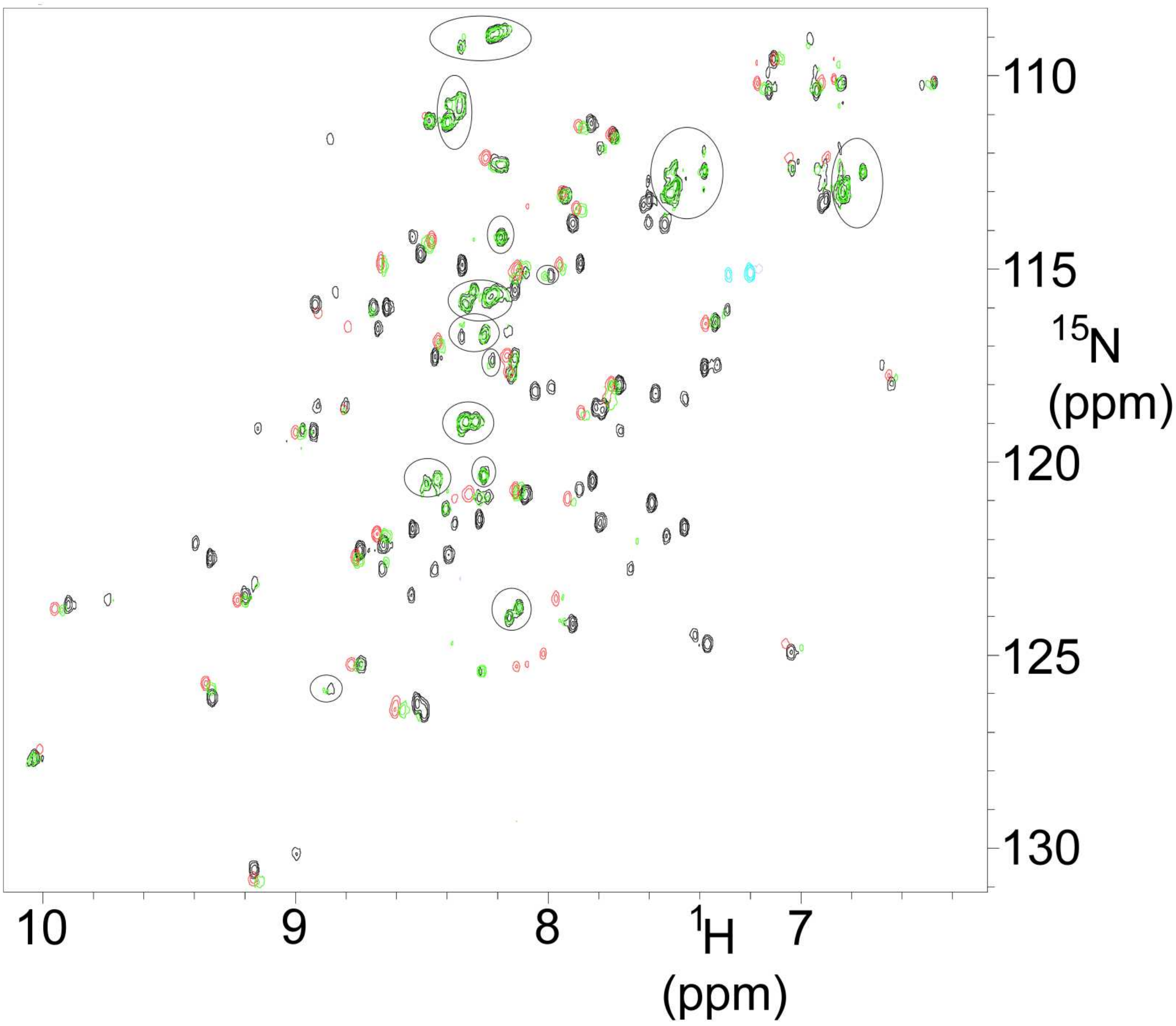

Supplementary Figure 6. HSQC spectra of 1L2L3 (black), 1L2L3 plus ca. 8 equivalents of GlcNAc$_6$ (green), and 1 plus 7 equivalents of GlcNAc$_6$ (red). All proteins are approximately 50 μM. Signals in cyan are from arginine sidechains. Signals with ellipses drawn round them are from residues in linkers and therefore do not have corresponding signals in module 1. For the other signals, there is an excellent correspondence in all three LysM modules between the behaviour of 1L2L3 and 1: in other words, the green and red signals match closely (except for the signals with ellipses round them).

1. <u>Peptidoglycan</u> (β-1,4–GlcNAc-MurNAc-peptide polymer)

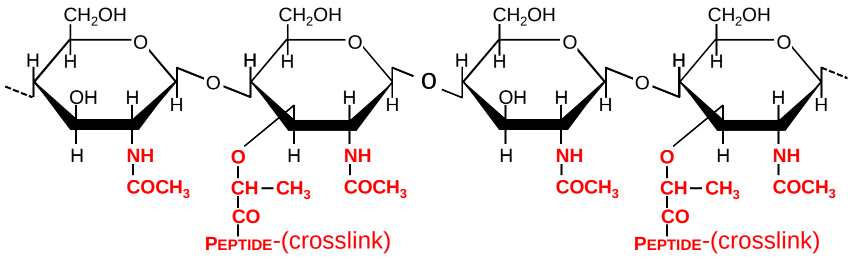

2. <u>Chitin</u> (β-1,4–GlcNAc polymer)

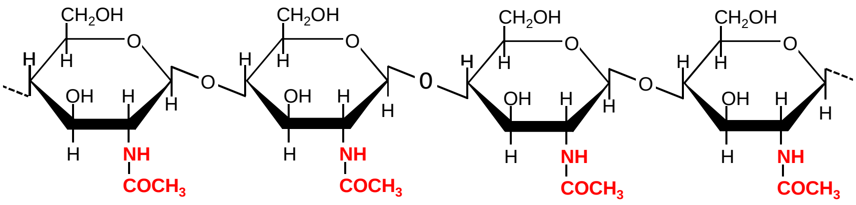

3. <u>Cellulose</u> (β-1,4–Glucose polymer)

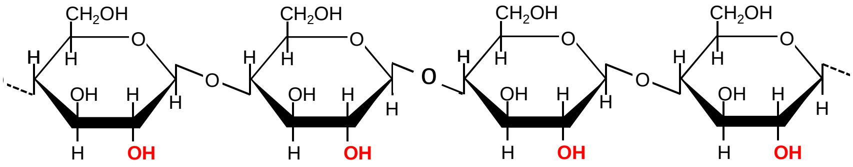

4. <u>Xylan</u> (β-1,4-xylose polymer)

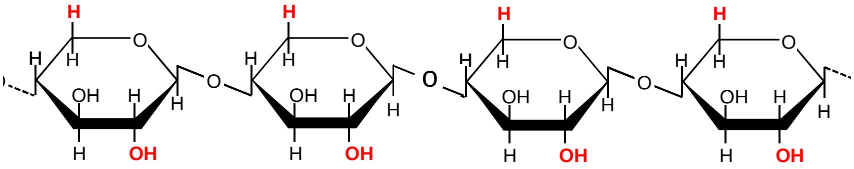

Supplementary Figure 7. Structures of the polysaccharides used for LysM affinity purification. The differences between the polymers are highlighted in red.

a

Immobilisation Concentration (µM)	RU immobilized	Affinity constants (K_D) at various flow rates		
		10µl/min	25µl/min	65µl/min
15.625	1605	12.35 ± 1.4	ND	ND
3.125	1484	10.57 ± 1.14	9.98 ± 1.13	10.59 ± 1.07
0.625	1423	10.51 ± 1.00	10.42 ± 1.13	10.69 ± 1.18
0.0625	484	26.35 ± 2.78	20.22 ± 2.02	21.63 ± 2.11

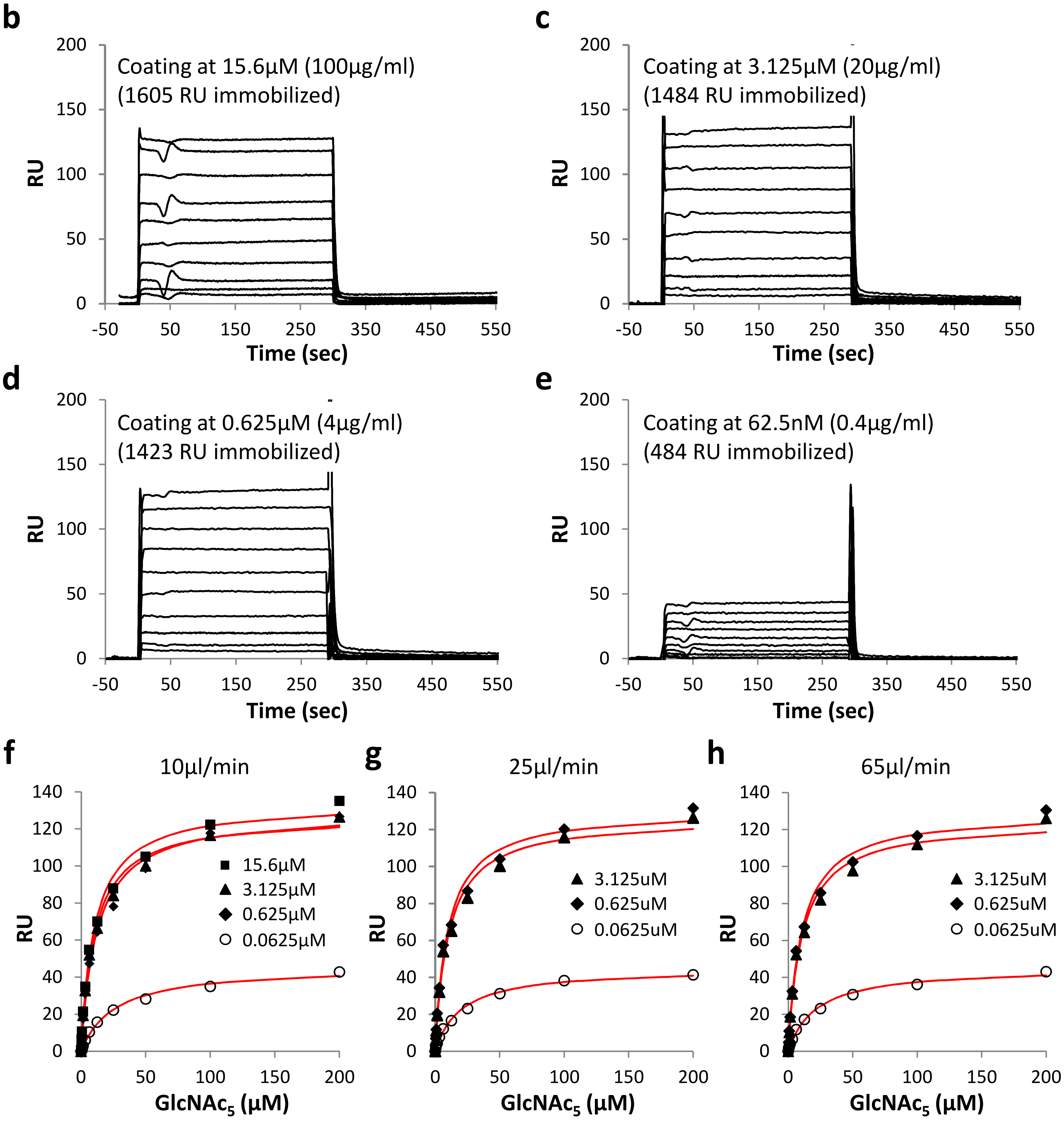

Supplementary Figure 8. Analysis of LysM-chitooligosaccharide interactions by surface plasmon resonance (SPR). Equilibrium affinity constants (K_D) were determined under steady-state conditions using $GlcNAc_5$ as analyte and 1LysM. (**a**) Impact of protein concentration and flow rate on affinity measurement. Typical sensorgrams obtained with $GlcNAc_5$ concentrations ranging from 200 µM to 0.39 µM are shown for each protein concentration used for immobilization: 15.625 µM (**b**); 3.125 µM (**c**); 0.625 µM (**d**); 0.0625 µM (**e**). Titration curves at various flow rates: 10 µl min^{-1}, (**f**); 25 µl min^{-1}, (**g**) 65 µl min^{-1}, (**h**). RU, resonance units . These results showed very limited mass transfer under all conditions tested and consistent K_D measurements at high density immobilization (protein concentration >4 µl ml^{-1}).

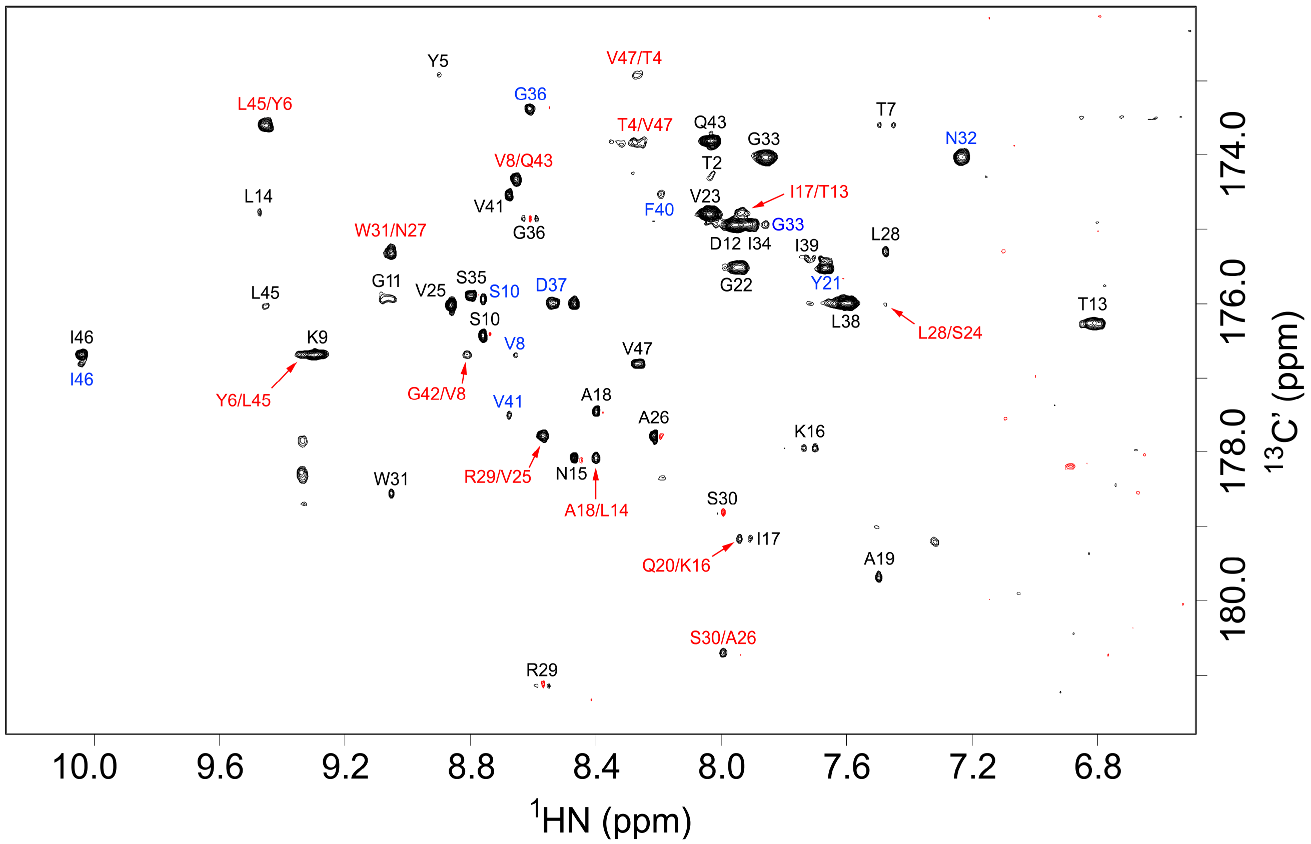

Supplementary Figure 9. Long-range H(N)CO experiment used to determine the locations of backbone hydrogen bonds. Hydrogen bond partners are indicated in red, intraresidue (three-bond) connections in blue and sequential (two-bond) connections in black.

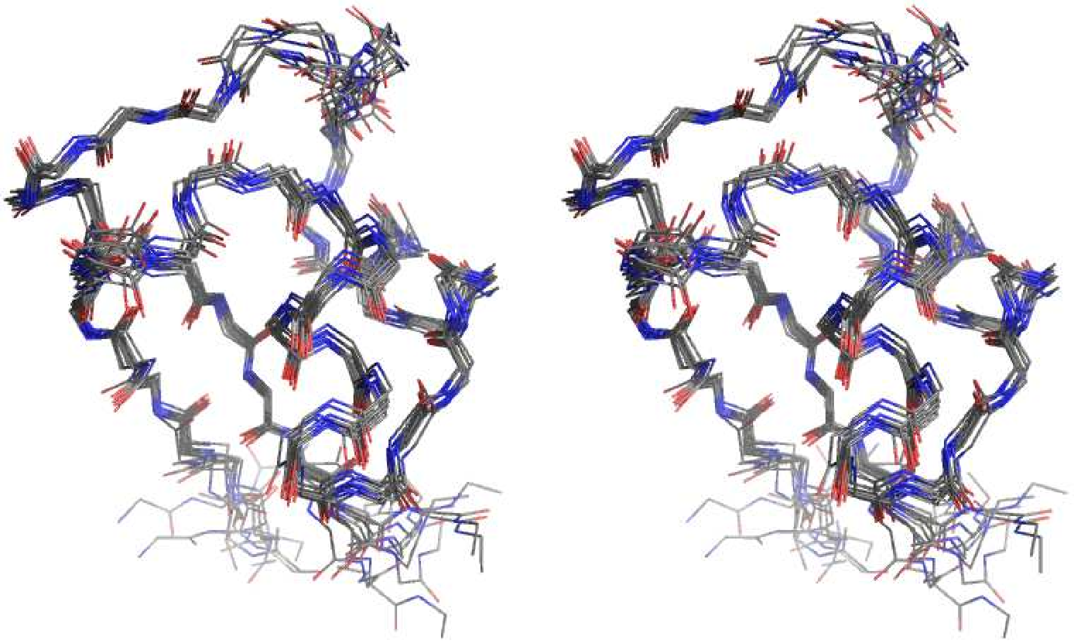

Supplementary Figure 10. Stereo view of the best superimposition of the backbone atoms of the 10 lowest energy NMR structures of the LysM domain.

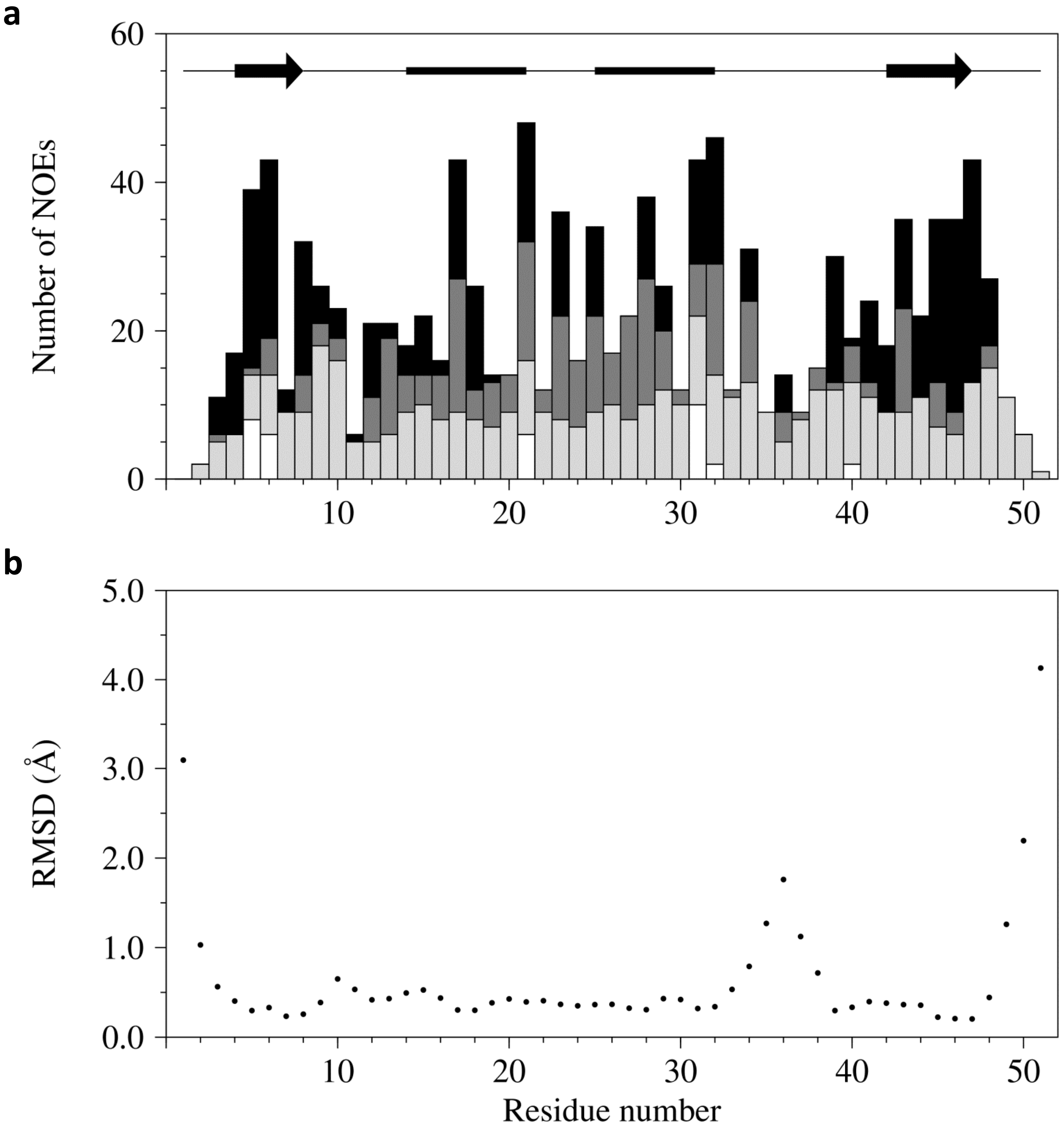

Supplementary Figure 11. Structural parameters for AtlA 1LysM. (**a**) Distribution (number) of NOE restraints by residue coloured according to NOE type; intraresidue (white), sequential (light grey), medium-range (dark grey) and long-range (black). (**b**) RMS deviations (RMSD) from the average structure for backbone atoms N, Ca and C', plotted as an averaged data point for each residue.

a

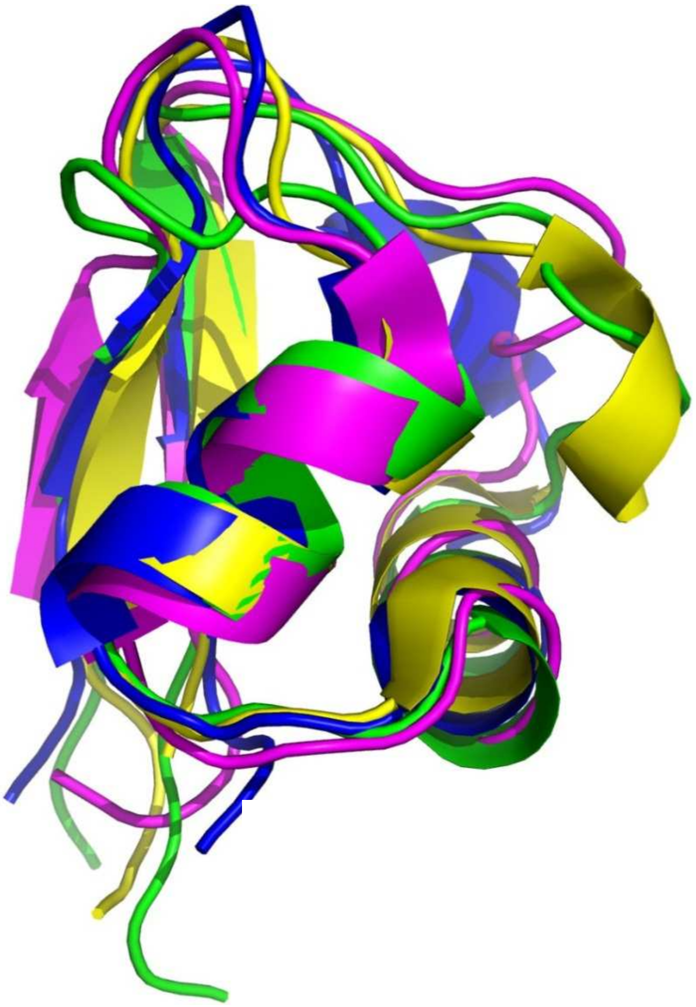

b

```
                10        20        30        40        50
       ....|....|....|....|....|....|....|....|....|....|.
1LysM  GTNTYYTVKSGDTLNKIAAQYGVSVANLRSWN-GISGDLI-FVQKLIVKK-
1oeg   -DSITYRVRKGDSLSSIAKRHGVNIKDVMRWN-SDTANLQ-PGDKLTLFVK
1y7m   ---LTYQVKQGDTLNSIAADFRISTAALLQANPSLQAGLT-AGQSIVIPGL
2l9y   --TATVTVQQGDTLRDIGRRFDCDFHEIARRNNIQNEDLIYPGQVLQVP--
```

Supplementary Figure 12. Structural alignment of LysM modules . (**a**) Superposition and sequence alignment of the NMR structure of 1LysM (2mkx; green) with the *E. coli* MltD (1e0g; blue), *B. subtilis* YkuD (1y7m; magenta) and the LysM domain from the type III lectin from fungus *Magnaporthe oryzae* (2l9y; yellow). Backbone RMSDs to 1LysM are respectively 1.47, 1.47 and 1.19 Å. (**b**) Amino acid sequence alignments of the 4 LysM modules shown in (**a**). Residues boxed in black indicate at least 75% conservation, and residues boxed in light grey indicate conservative substitutions.

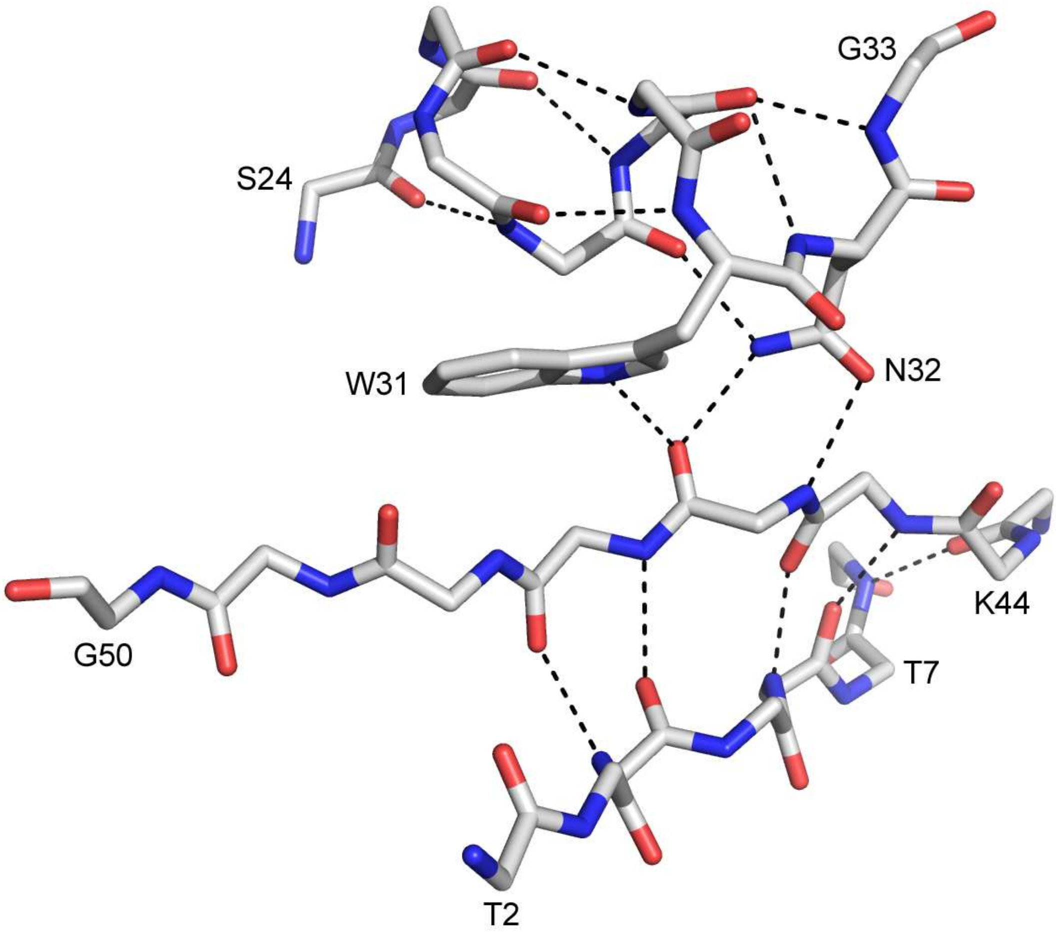

Supplementary Figure 13. Part of the 1LysM structure showing secondary structure hydrogen bond networks. Residues W31 and N32 are involved both in a helix capping arrangement for a-helix V25-N32 and in the coordination of the antiparallel b-strand G42-V47. Such an arrangement is confirmed by the significant downfield chemical shift values for the sidechain NH_2 group of N32 (8.895 and 9.032 ppm) which coordinate the backbone carbonyl groups of I46 and L28. Also, the electronic shielding effect of the W31 ring perturbs the backbone HN group of K48 such that it is upfield shifted to 6.778 ppm.

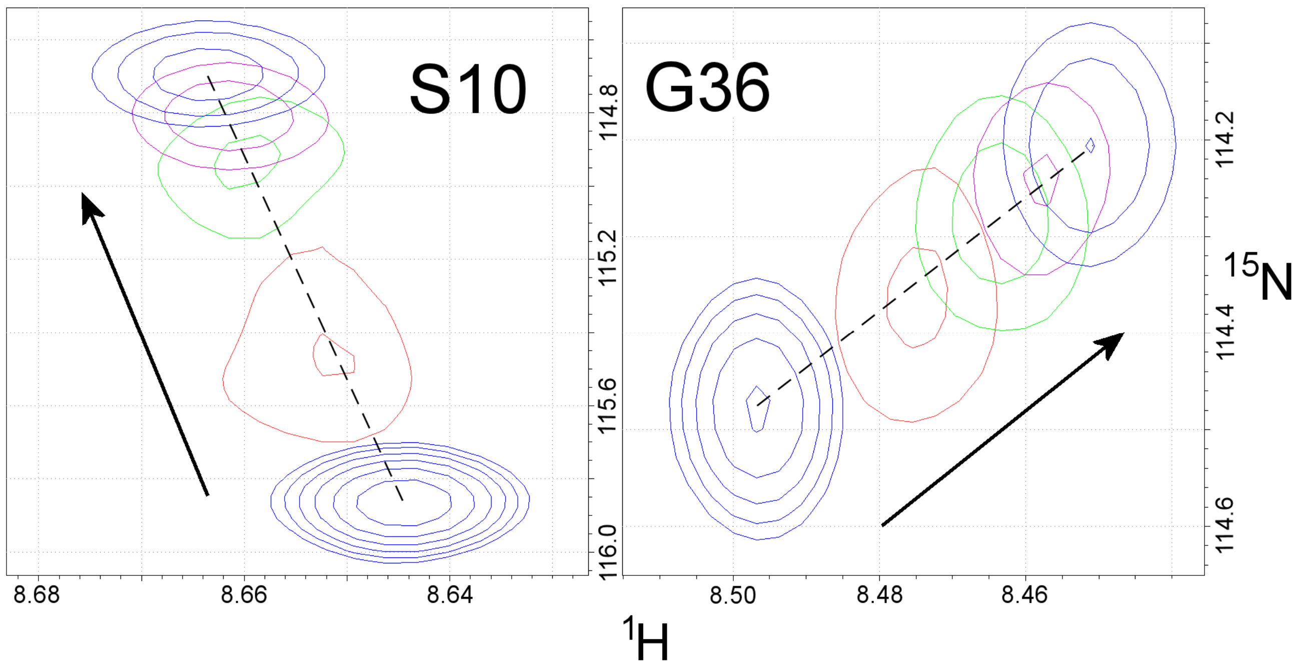

Supplementary Figure 14. NMR titration of 1LysM with $GlcNAc_5$. Residues S10 and G36 are shown. The titration used 50 µM protein, and shows the addition of 0 (blue), 1.5 (red), 4 (green), 8 (magenta) and 23.6 (blue) equivalents of ligand. The contour spacing is the same for all peaks, showing line broadening (broader and weaker peaks in the presence of ligand). The dashed lines show the expected locations of peak centres in the absence of exchange.

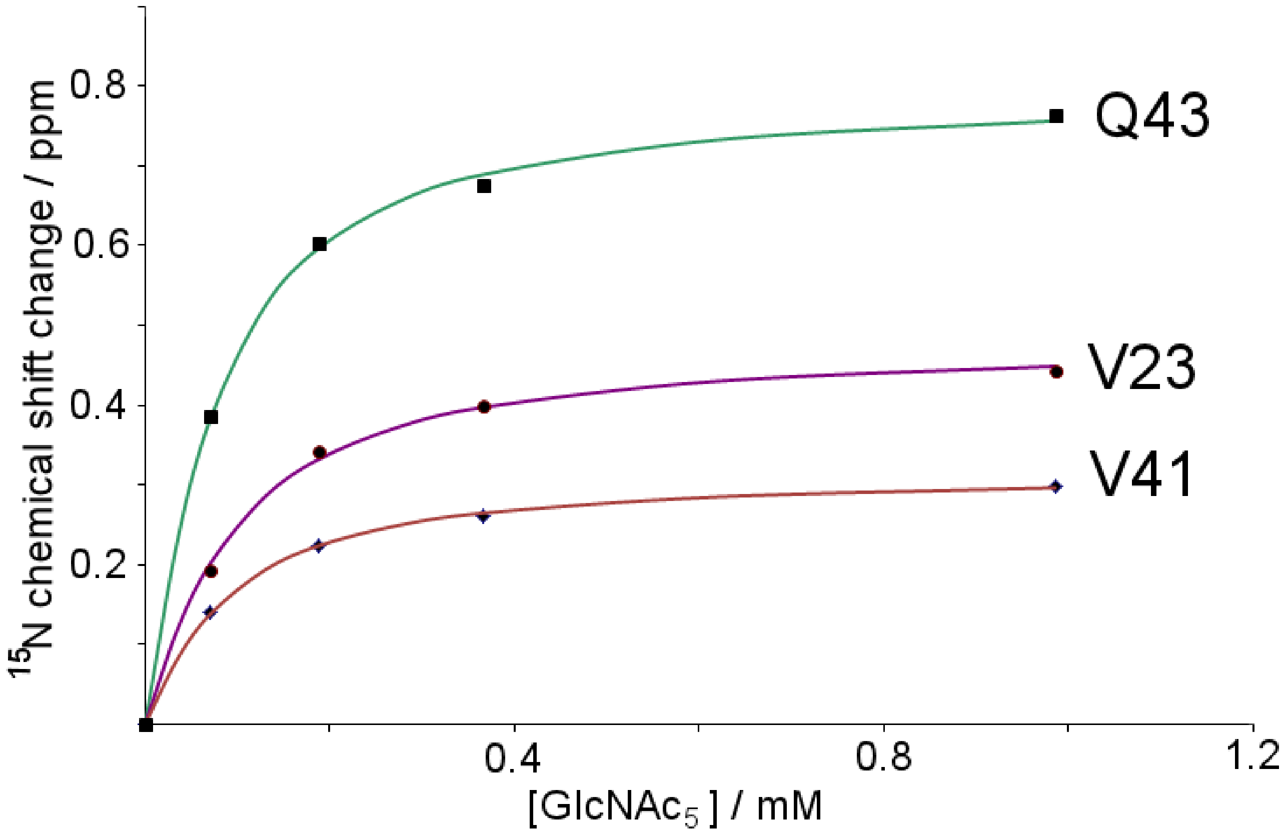

Supplementary Figure 15. NMR titration of 1LysM with $GlcNAc_5$, showing ^{15}N chemical shift changes of residues Q43 (■, green curve), V23 (●, blue curve) and V41 (◆, pink curve) with fitted curves.

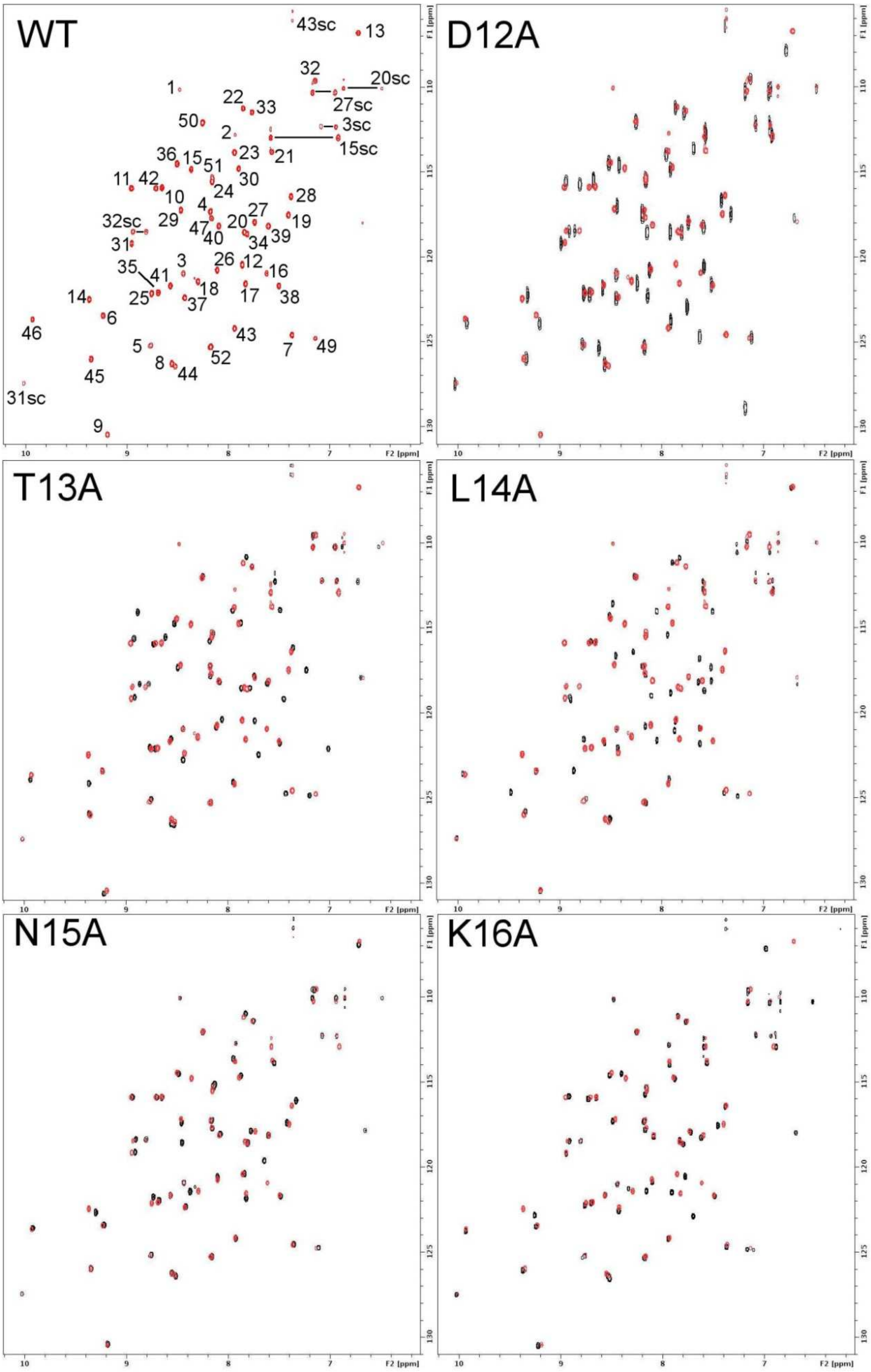
WT
D12A
T13A
L14A
N15A
K16A
43sc
13
32
20sc
1
22
33
27sc
50
2
3sc
15sc
36
15
23
21
51
11
42
30
28
24
10
4
27
32sc
29
47
20
19
40
39
31
34
26
12
3
35
16
41
18
25
17
38
14
37
6
46
43
5
7
49
52
8
44
45
31sc
9
F1 [ppm]
F2 [ppm]
10
9
8
7
110
115
120
125
130

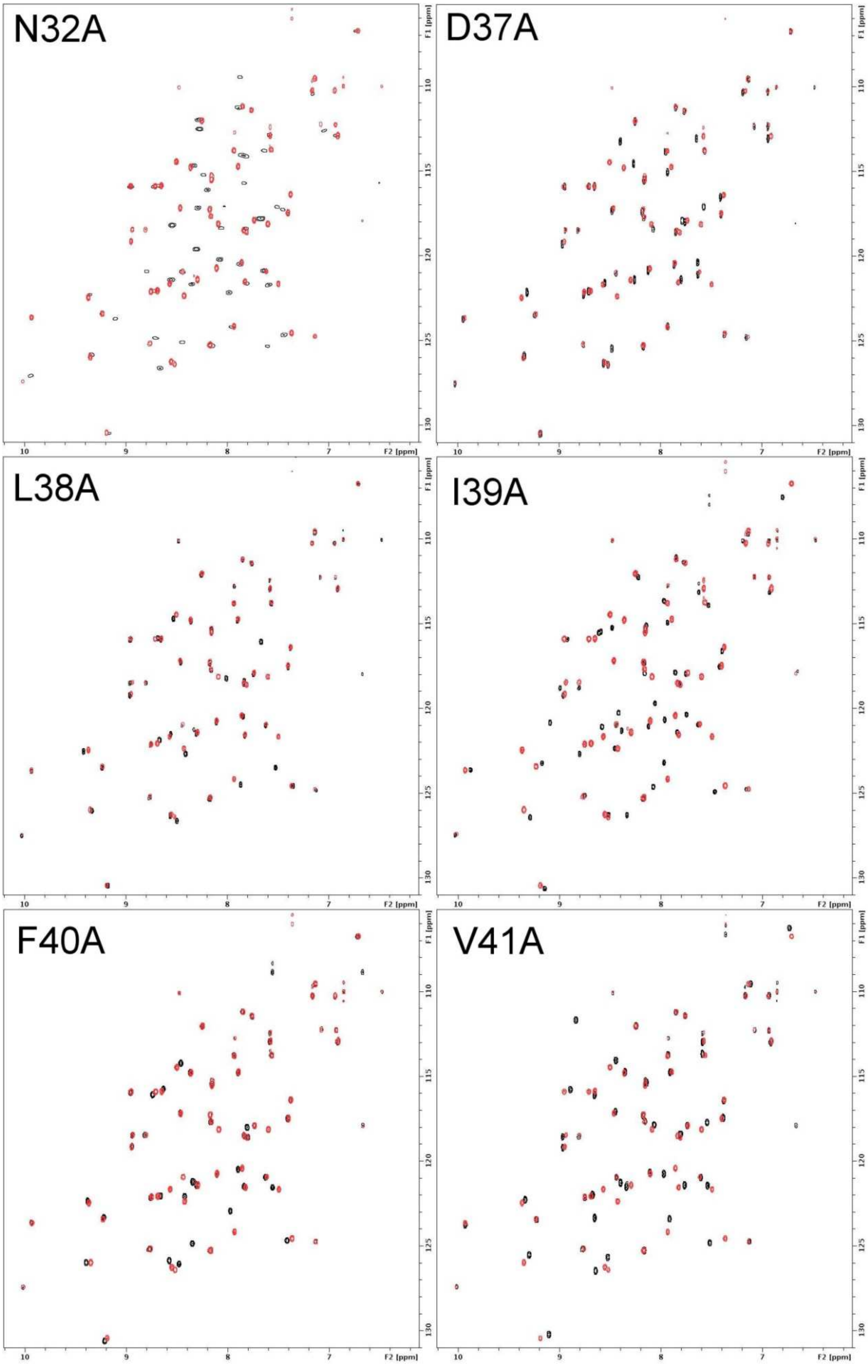

Supplementary Figure 16. ^{15}N HSQC spectra of 1LysM and the 11 single-site alanine mutants studied by NMR. All spectra are 50 μM protein in 40 mM phosphate buffer pH 6.0. For each mutant, the WT spectrum (red) is superimposed on top of the mutant spectrum (black).

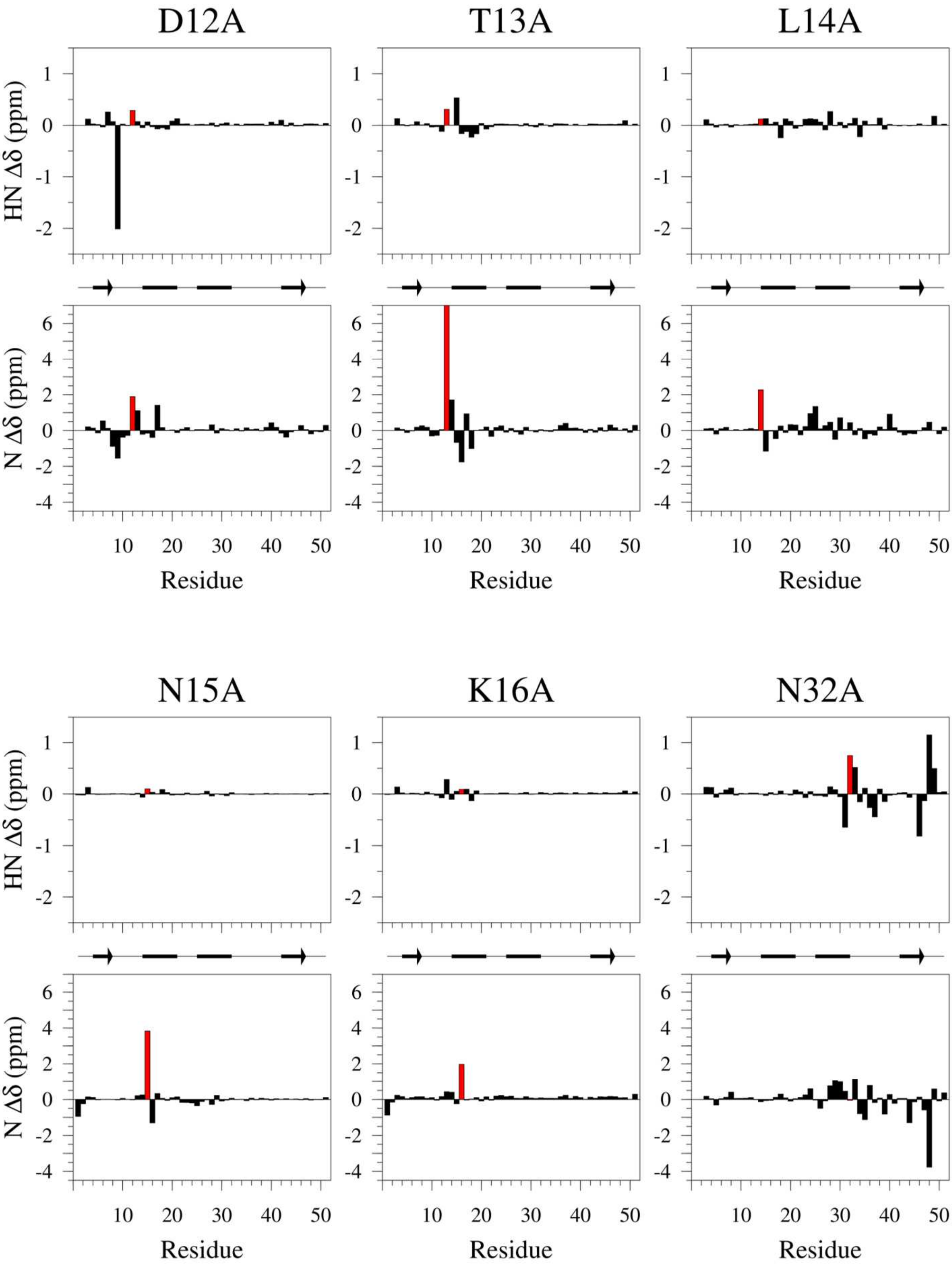

D12A
T13A
L14A
N15A
K16A
N32A
HN Δδ (ppm)
N Δδ (ppm)
Residue

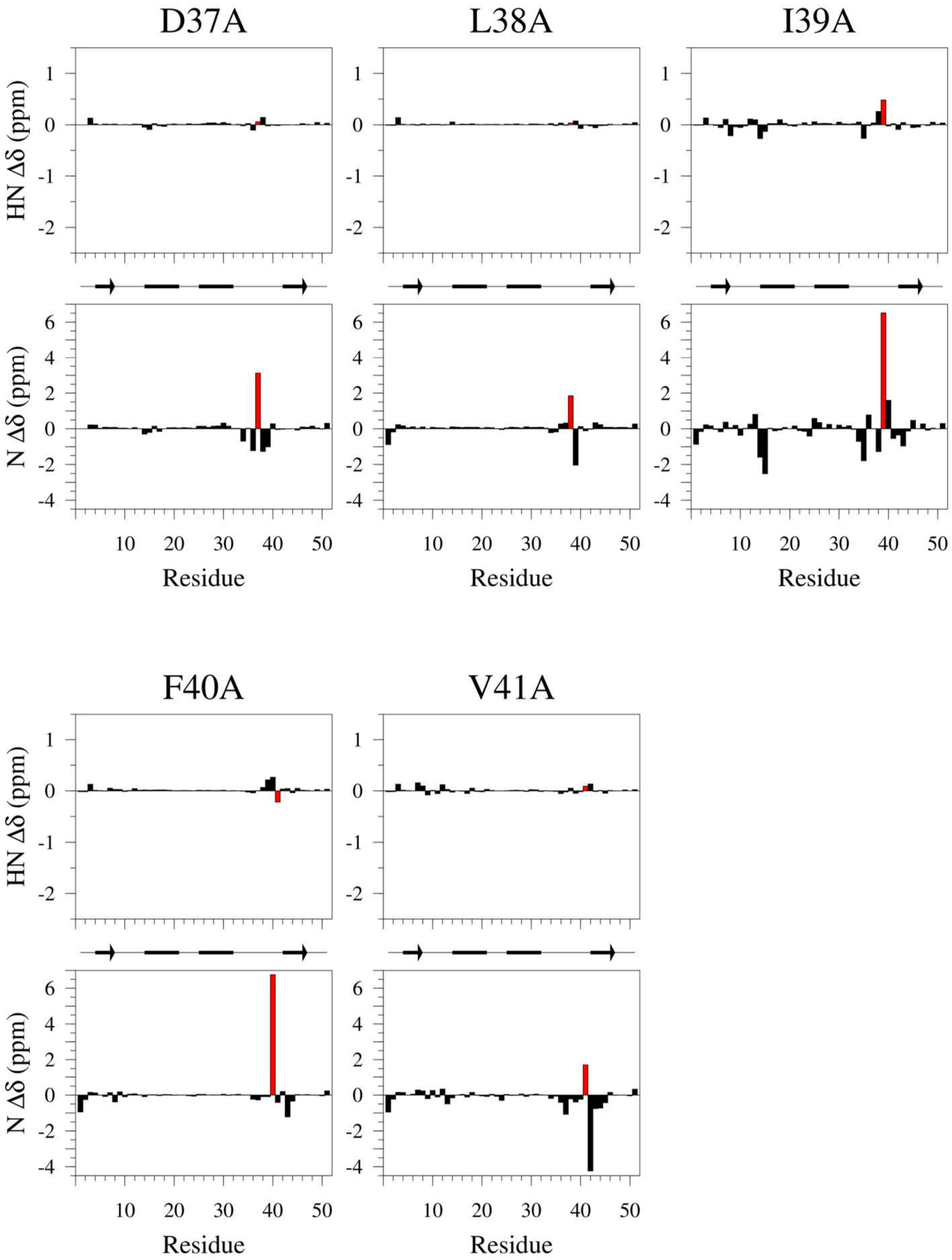

Supplementary Figure 17. Analysis of HN and N chemical shift changes between WT and mutant for the single-site alanine mutants defined as [δ_{mutant} - δ_{WT}]. The mutated residue is shown in red. Small chemical shift changes show that all mutants are correctly folded. The largest disruption to the native fold is seen for I39A (part of the hydrophobic core) and N32A (N32 sidechain is buried and is hydrogen bonded to two backbone carbonyls). For T13A the ^{15}N chemical shift change of residue 13 is 15.4 ppm.

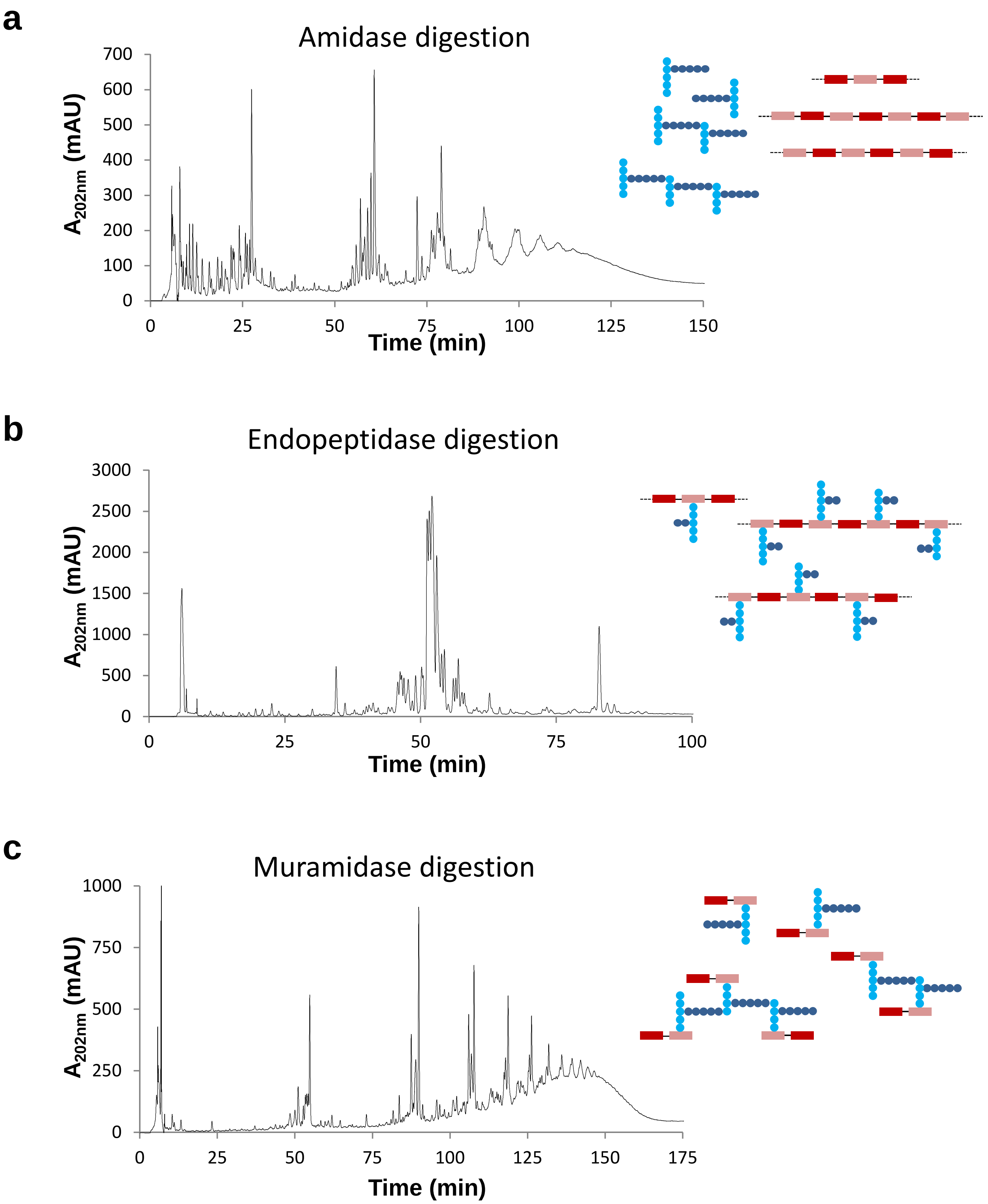

Supplementary Figure 18. rp-HPLC muropeptide profiles of *S. aureus* peptidoglycan fragments used for SPR analyses. Prior to injection, soluble peptidoglycan fragments were reduced with sodium borohydride. An aliquot corresponding to 200 µg of peptidoglycan was injected on a Hypersil GOLD aQ column (C18; 2.1 by 200 mm, Thermo Scientific) and separated at a flow rate of 0.5 ml min^{-1} using 10 mM ammonium phosphate (pH 5.5) as a mobile phase (buffer A). After a 10 min isocratic step, muropeptides were eluted with a 270 min methanol gradient (0 to 30% linear gradient in buffer A). **(a)** Amidase digestion. (**b**) endopeptidase (lysostaphin) digestion. (**c**) muramidase (mutanolysin) digestion.

SUPPLEMENTARY TABLES

Supplementary Table 1. Apparent binding affinities of 1LysM module to various substrates. Apparent K_D values determined by SPR and NMR are in μM ± standard deviation.

**Complex**	**SPR**	**NMR**
1LysM + $GlcNAc_6$	6.0 ± 0.8	110 ± 30
1LysM + $GlcNAc_5$ [a]	12.3 ± 1.4	66 ± 11
1LysM + $GlcNAc_4$	43.4 ± 3.0	90 ± 20
1LysM + $GlcNAc_3$	407.9 ± 80.8	710 ± 30
1LysM + pentachitosan	-[b]	ND[c]
1LysM + xylan	-[b]	ND[c]
1LysM + cellulose	-[b]	ND[c]
1LysM + dextran	ND[c]	-[b]
1LysM + carboxymethyl dextran	ND[c]	-[b]
1LysM + dextran sulphate	ND[c]	-[b]
1LysM + heparin	ND[c]	-[b]

[a] K_D = 92 ± 2.5 μM as determined by Isothermal Titration Calorimetry

[b] -, not tested

[c] ND, Not detectable

Supplementary Table 2. Structural parameters for AtlA 1LysM [a] .

Parameter	Value
Experimentally determined restraints	
Total unambiguous distance restraints[b]	430
Intraresidue	14
Sequential	168
Medium range	106
Long range ($\Delta > 4$)	142
Dihedral angle restraints[c]	98
Hydrogen bond restraints	16×2
Deviations from the experimental restraints	
RMS NOE restraint violation (Å)	0.037 ± 0.001
NOE restraint violations > 0.0 Å	57.0 ± 4.7
Maximum NOE restraint violation (Å)	0.3
RMS dihedral restraint violation (°)	1.489 ± 0.077
Dihedral restraint violations > 0.0001°	33.2 ± 2.4
Maximum dihedral restraint violation (°)	10.1
Mean RMS deviations from idealised geometry	
Bond lengths (Å)	0.0045 ± 0.0002
Angle geometry (°)	0.694 ± 0.027
Improper geometry (°)	2.000 ± 0.125
RMS deviations from the mean structure (Å) for residues 3–49	
Backbone heavy atoms	0.56
All heavy atoms	0.96
Ramachandran analysis[d]	
Most favoured regions (%)	89.0
Additionally allowed regions (%)	11.0
Generously allowed regions (%)	0.0
Disallowed regions (%)	0.0

[a] Ensemble of the 10 lowest energy structures out of a total of 100 calculated structures.

[b] NOE cross-peak intensities were divided into three groups with upper bounds of 2.8, 3.4 and 6.0 Å.

[c] Dihedral angle restraints were implemented with a range of ± 2 standard deviations (φ and ψ) from TALOS-N or a range of ± 30° (χ^1).

[d] PROCHECK-NMR [1] analysis of residues 3–49 of the structural ensemble

Supplementary Table 3. Bacterial strains and plasmids used in this study.

Strain/plasmid	Relevant properties	Source/reference
E. coli strains		
Top10F'	Cloning strain; F´ {*lacI*^{q} *Tn10* (Tet^R)*}*^{2} *mcrA* Δ*(mrr-hsdRMS-mcrBC)* *ϕ80*Δ*(lacZ)M15* Δ*lacX74 deoR recA1 araD139* Δ*(ara-leu)7697 galU galK rpsL* (Str^R) *endA1 nupG*	Invitrogen
C43(DE3)	BL21(DE3) derivative used as an expression strain for LysM domains	Lucigen
Plasmids		
pET2818	pET derivative for expression of proteins with a C-terminal 6-His tag	[2]
pML520	pET derivative for the expression of recombinant LysM polypeptide 1	this study
pML519	pET derivative for the expression of recombinant LysM polypeptide L1	this study
pML521	pET derivative for the expression of recombinant LysM polypeptide 1L2	this study
pML523	pET derivative for the expression of recombinant LysM polypeptide L1L2	this study
pML522	pET derivative for the expression of recombinant LysM polypeptide 1L2L3	this study
pML524	pET derivative for the expression of recombinant LysM polypeptide L1L2L3	this study
pML5180	pET derivative for the expression of recombinant LysM polypeptide L1-L6	this study

Supplementary references

1. Laskowski RA, Rullmannn JA, MacArthur MW, Kaptein R, Thornton JM. AQUA and PROCHECK-NMR: programs for checking the quality of protein structures solved by NMR. *Journal of biomolecular NMR* 1996, 8(4): 477-486.

2. Mainardi JL, Fourgeaud M, Hugonnet JE, Dubost L, Brouard JP, Ouazzani J, *et al.* A novel peptidoglycan cross-linking enzyme for a beta-lactam-resistant transpeptidation pathway. *The Journal of biological chemistry* 2005, 280(46): 38146-38152.
